# An optimised patient-derived explant platform for breast cancer reflects clinical responses to chemotherapy and antibody-directed therapy

**DOI:** 10.1038/s41598-024-63170-0

**Published:** 2024-06-04

**Authors:** Constantinos Demetriou, Naila Abid, Michael Butterworth, Larissa Lezina, Pavandeep Sandhu, Lynne Howells, Ian R. Powley, James H. Pringle, Zahirah Sidat, Omar Qassid, Dave Purnell, Monika Kaushik, Kaitlin Duckworth, Helen Hartshorn, Anne Thomas, Jacqui A. Shaw, Marion MacFarlane, Catrin Pritchard, Gareth J. Miles

**Affiliations:** 1 Leicester Cancer Research Centre, University of Leicester, Clinical Sciences Building, Leicester, LE2 7LX UK; 2 HOPE Clinical Trials Facility, University Hospitals of Leicester NHS Trust, Sandringham Building, Leicester Royal Infirmary, Leicester, LE1 5WW UK; 3 Pathology Department, University Hospitals of Leicester NHS Trust, Leicester Glenfield General Hospital, Groby Road, Leicester, LE3 9QP UK; 4 Breast Care Centre, University Hospitals of Leicester NHS Trust, Leicester Glenfield General Hospital, Groby Road, Leicester, LE3 9QP UK; 5 MRC Toxicology Unit, Gleeson Building, Tennis Court Road, Cambridge, CB2 1QR UK; 6 Department of Molecular and Cell Biology, University of Leicester, Leicester, LE1 7HB UK

**Keywords:** Patient-derived explants, Therapies, HER2, Breast cancer, Multi-immunofluorescence, Cancer, Breast cancer, Experimental models of disease

## Abstract

Breast Cancer is the most common cancer among women globally. Despite significant improvements in overall survival, many tumours are refractory to therapy and so novel approaches are required to improve patient outcomes. We have evaluated patient-derived explants (PDEs) as a novel preclinical platform for breast cancer (BC) and implemented cutting-edge digital pathology and multi-immunofluorescent approaches for investigating biomarker changes in both tumour and stromal areas at endpoint. Short-term culture of intact fragments of BCs as PDEs retained an intact immune microenvironment, and tumour architecture was augmented by the inclusion of autologous serum in the culture media. Cell death/proliferation responses to FET chemotherapy in BC-PDEs correlated significantly with BC patient progression-free survival (*p* = 0.012 and *p* = 0.0041, respectively) and cell death responses to the HER2 antibody therapy trastuzumab correlated significantly with HER2 status (*p* = 0.018). These studies show that the PDE platform combined with digital pathology is a robust preclinical approach for informing clinical responses to chemotherapy and antibody-directed therapies in breast cancer. Furthermore, since BC-PDEs retain an intact tumour architecture over the short-term, they facilitate the preclinical testing of anti-cancer agents targeting the tumour microenvironment.

## Introduction

Among women, breast cancer is the most common global cancer, with 2.1 million cases diagnosed annually^1^. Despite significant advances in early diagnosis and treatments for breast cancer resulting in 5-year overall survival at 80% globally^2^, breast cancer continues to contribute 15% to overall cancer mortality in women^1^. In the era of personalised medicine, coupled to breakthroughs in molecular biology and immunotherapy, specific targeted therapies have been developed for different pathophysiological types of breast cancer. For example, oestrogen-dependent tumours are treated with selective oestrogen receptor modulators (SERMs) or aromatase inhibitors^3,4^ and the recombinant antibody trastuzumab is tailored for action against HER2-positive breast cancers^5,6^. Advanced metastatic breast cancer can be treated with bevacizumab, targeted against vascular endothelial growth factor (VEGF) in combination with taxanes^7^. Additionally, a plethora of clinical trials are under way to evaluate the efficacy of immunotherapies in patients with breast cancer^8^. Despite these tremendous successes, a subset of tumours is refractory to treatment. Therefore, more accurate and cost-effective methods for predicting therapeutic benefit within a clinically-relevant window are required to improve clinical outcomes.

Common endpoints of drug efficacy at the preclinical level often rely on cell line-derived xenografts, which are not always predictive of compound efficacy in the clinical setting^9^. Whilst patient-derived xenograft (PDX) mouse studies have increased predictivity in the clinic^10,11^, these models are expensive, take a long time to propagate and not all human tumours can be generated into PDX mice, with those that do often losing human tumour characteristics with serial propagation. Use of patient-derived cancer organoids has been widely explored as suitable alternatives for predictors of treatment response^12^ in multiple solid tumour types and are used extensively in breast cancer research^13^. However, current organoid models have the drawback in that they are derived from deconstructed tumours and are therefore^14^ limited in their ability to faithfully recapitulate the patient-specific tumour ecosystem and microenvironment.

Alternative methods of using fresh breast tumour organ cultures or breast cancer explants have been implemented for the assessment of therapeutic responses, with varying degrees of success. Breast cancer tissue was first maintained in culture by Cameron & colleagues^15^ for 2–4 months with these epithelial cellular nodule outgrowths forming luminal structures. Later studies^16^ demonstrated that protracted culture resulted in spilled-out epithelial islands distal to the explants, which contained no stromal content, raising the question of whether the tumour microenvironment is maintained in extended culture. Another drawback of previous breast explant studies is that, typically, endpoint analysis involved manual scoring of cells or biomarkers without any focus on the spatial distribution of drug response^17–19^.

We have recently reported the culture of Non-Small Cell Lung Cancer (NSCLC) and Endometrial Cancer (EC) patient-derived explants (PDEs) that involve the short-term culture of 3D fragments of freshly resected human tumours at the air–liquid interface^20,21^. Our work with NSCLC-PDEs confirmed a significant relationship with PDE responses to chemotherapy drugs and patient outcomes, and the clinical predictivity of the platform has also been confirmed through similar work by others for colorectal cancer (CRC)^22^. Here, we have adapted the methodology for the culture of breast cancer PDEs (BC-PDEs). Unlike previous longer-term breast explant cultures, we show that the tumour-stroma architecture is maintained intact using this short-term culture method and that drug responses can be assessed in both tumour and stroma areas. We implement an advanced multiplexed immunofluorescence (mIF) and whole slide scanning method, coupled with digital pathology solutions to undertake spatial profiling of biomarker expression at endpoint. The predictive power of this platform is investigated by assessing BC-PDE responses to a typical standard-of-care regime of 5-fluorouracil, epirubicin, and docetaxel and to the HER2 antibody-directed therapy trastuzumab, with our data showing that BC-PDE responses are consistently reflective of clinical parameters. We also evaluate for the first time the BC-PDE treatment responses in non-tumour stromal tissue.

## Methods

### Patient samples

Freshly resected Breast Cancer (BC) tumours (> 25 mm in size), and pre-operative bloods were collected from 55 consented patients (Ethical approval REC: 14/WA/1166) undergoing breast cancer surgery at the Leicester Glenfield General Hospital, University Hospitals of Leicester NHS Trust (UHL). Details of adjuvant therapy given to each patient are provided in Additional File 1. Out of the 55 patients, 6 received neo-adjuvant endocrine therapy, and these are also indicated in Additional File 1. Diagnostic reports were obtained from consultant pathologists at University Hospitals of Leicester, UK and molecular subtypes were assigned based on the following criteria: Luminal A: High ER (7/8), HER2 negative, Ki67 < 14%; Luminal B: High ER (7/8), HER2 negative, Ki67 > 14%; TNBC: Low ER (0–3), HER2 negative; HER2-Enriched: HER2 positive irrespective of ER/PR positivity. HER2 positive was either(1) HER2 with IHC score of 3 + ; (2) HER2 with IHC score of 2 + and HER2 gene amplification positive as determined by FISH and reported in the histopathology reports and, (3) where available, copy number gains in HER2 as determined by whole exome sequencing analysis of the primary tumour. ER, PR and HER2 status were extracted from post-surgical pathology reports.

### Autologous serum

7.5 mL pre-operative blood was collected in S-Monovette (Sarsedt) serum collection tubes and centrifuged immediately at 1000 *g* at 20°C for 10 min. Serum was separated from coagulated red blood cells and stored at − 80 °C until required.

### Patient-derived explant (PDE) culture

The methodology for PDE generation from fresh tumours is described in detail^23^. Breast cancer patients undergoing mastectomy or wide local excisions with tumours > 25 mm were recruited to the study. Excess surplus tumour tissue was provided by consultant pathologists, transported to the laboratory on ice in holding media (Phenol-free DMEM-F12) and processed to PDEs within 2 h. Fat and obvious necrotic areas were removed then the tumour tissue was manually diced on a wax dental plate (Anūtex) into 2–3 mm^3^ fragments using skin graft blades. After derivation, 6–9 PDEs per patient sample were randomly selected and fixed in 4% (w/v) paraformaldehyde immediately for base line (T0) control to reflect original tissue properties/architecture.

For media optimisation, 6–9 PDEs were randomly selected and placed on a Millicell 0.4 µm 30 mm PTFE culture insert disc (Millipore, PICM0RG50), and floated in a 6 well plate in 1.5 mL of 11 defined media conditions for 16–20 h overnight to recover at 37 °C and 5% CO_2_ in a humidified atmosphere followed by a further 24 h in fresh media. Exact media formulations are detailed in Additional File 2.

For standard-of-care (SoC) drug treatments, after the recovery period, PDEs were incubated for 24 h in 1.5 mL fresh treatment media with or without 1.15 μM 5-Fluorouracil (Sigma), 0.1 μM epirubicin, 3 μM docetaxel ± 5 μg/mL Trastuzumab. 0.15% (v/v) dimethysulphoxide (DMSO) was used as a vehicle control. After culture, PTFE inserts containing PDEs were floated on 1.5 mL 4% (w/v) paraformaldehyde for 24 h. After fixation they were transferred onto histology sponges, placed in histology cassettes and then embedded in paraffin wax blocks.

### Histological analysis and multiplexed immunofluorescence (mIF)

H&E staining of formalin fixed paraffin-embedded (FFPE) 4 μm sections were generated using standard approaches^24^. Akoya Biosciences Tyramide signal amplification (TSA) was used for multiplexed immunofluorescence (mIF) to visualise multiple biomarkers on the same slide as previously published^23,25^ and using the manufacturer’s instructions. Details of all antibodies used, the incubation times and fluorophore pairs are detailed in Additional File 3. After the final Opal fluorophore in each mIF panel, slides were washed in NP water then incubated with 6 µM DAPI for 5 min, washed in NP water before mounting in ProLong Diamond Antifade mountant (Life Technologies). For an autofluorescent (AF) control slide, the same staining procedure was applied to an additional section, omitting all antibodies/fluorophores/DAPI. Stained whole slides were digitised using an Akoya Biosciences Vectra Polaris in whole slide MOTIF imaging mode using a 20 × objective lens, and excitation and emission filters: Opal-480, Opal-520, Opal-570, Opal-690, AF and DAPI.

### Image analysis

Digitised whole-slide scans were visualised using Phenochart (Akoya Biosciences), where PDEs were selected for further image analysis. Images were imported into the InForm Image analysis software (Akoya Biosciences Version 2.5). The AF slide was used to remove all AF from the analysis slides using the AF picker tool in InForm, by selecting areas of autofluorescent tissue and avoiding background glass slide. InForm was taught by example to segment the PDEs into four tissue categories: Tumour, Stroma, Necrosis and Background/glass. Tumour was defined by the operator as cytokeratin^+ve^/DAPI^+ve^, stroma was defined as cytokeratin^-ve^/DAPI^+ve^, and necrosis was defined as cPARP^+ve^/DAPI^-ve^ ± cytokeratin. In mIF panels where there was no cPARP staining tissue categories were defined as Tumour, Stroma or background using the above classifications. Individual cells were identified using InForm’s adaptive cell segmentation algorithm to segment the individual nuclei based on DAPI staining. Settings were applied separately for each patient sample to achieve the optimal cell segmentation for each patient. Finally, InForm was trained to phenotype individual cells based on Opal fluorophore staining into specific phenotypes for each mIF Panel, using InForms Phenotyping algorithm.

For Ki67 and cPARP mIF panel, phenotypes were defined as: proliferating cells (Ki67^+ve^ DAPI^+ve^), apoptotic cells (cPARP^+ve^ DAPI^+ve^) and negative cells (DAPI^+ve^). For Ki67 and Geminin mIF panel, phenotypes were defined as: Geminin positive cells (Geminin^+ve^ Ki67^+ve^ DAPI^+ve^), Ki67 positive only cells (Ki67^+ve^ Geminin^-ve^ DAPI^+ve^) and negative cells (DAPI^+ve^). The operator could not identify Geminin^+ve^ Ki67^-ve^ DAPI^+ve^ only cells. For CD4, CD8, FOXP3 mIF panel, phenotypes were defined as T-effector (CD8^+ve^ DAPI^+ve^), T-helper (CD4^+ve^ FOXP3^-ve^ DAPI^+ve^), T-regulatory (CD4^+ve^ FOXP3^+ve^ DAPI^+ve^), Tumour (CK^+ve^ DAPI^+ve^) and negative cells (DAPI^+ve^).

### Calculation of apoptosis, necrosis and proliferation

Percent Ki67^+ve^ and cPARP^+ve^ cells was calculated for both tumour and stroma defined areas for every PDE from each treatment. Percent area of necrosis was calculated as a percentage area of each individual PDE. For intrinsic parameters the median value from T0 PDEs from each patient was used. For FET-induced percentage changes in viability, the median values of apoptosis and necrosis from FET treated samples were subtracted from median values derived from vehicle control treated values for each patient. For fold change drug response values, the median value for FET treated samples was divided by the median value for vehicle treated samples for each patient.

### Data and statistical analysis

Data and statistical analyses were performed using R for windows version 4.0.5. The packages tidyverse^26^ and Phenoptr (Akoya Biosciences) were used to tidy and wrangle the InForm outputs. The packages ggpubr and ggplot2 were used for statistical analysis. Unpaired data was evaluated for statistical significance using the Mann–Whitney test, *p* < 0.05 was considered significant. Linear correlations were calculated using Pearson’s Correlation. Trend tests were performed using Jonckheere-Terpstra trend test where *p* < 0.05 was deemed significant. R was used to generate an ROC curve test using pROC^27^, which was used to generate a threshold for PDE sensitivity/resistance. This was then used to define PDEs as sensitive/resistant to standard of care treatment, after which Kaplan–Meier plots were generated using packages survival and survminer where p < 0.05 was considered significant.

### Ethics approval and consent to participate

This was a fully consented, ethically approved study. The study was approved by the Wales REC 4 research ethics committee (REC ID: 14/WA/1166). The study was conducted in accordance with the Declaration of Helsinki. All participants gave informed consent for use of their cancer tissue samples.

## Results

In this study, 55 breast cancer patient samples were used, and their histopathological characteristics are summarised in Table 1. Invasive lobular and ductal carcinomas broadly matched expected distributions^28^. Interestingly, however, invasive mucinous carcinomas accounted for 12.7% of cases, which is sixfold higher than reported in the general patient population^28^. The reasons for this are not apparent at present. The distribution of molecular subtypes broadly matched typical distributions^28^. However, tumours were skewed towards higher grade/stage tumours than previously reported frequencies^29,30^, the most likely reason for this being that only patients exhibiting tumours > 25 mm were recruited to this study.

**Table 1:** Summary of patient variables and tumour clinicopathological variables, detailing histology, molecular subtype, stage, grade, and patient age.

Characteristic	Number (%) of patients/tumours
Number of patients	55
Age	
Median	66.5
Range	35–87
Histology	
Ductal	28 (50.9)
Lobular	8 (145)
Mucinous	7 (12.7)
Mixed	12 (21.8)
Subtypes	
Luminal A/Normal Like	36 (6.45
Luminal B	7 (12.7)
TNBC	8 (14.5)
HER2- Enriched	4 (7.3)
Stage	
IA	1 (1.9)
IB	2 (3.7)
IIA	24 (44.4)
IIB	11 (20.4)
IIIA	15 (27.8)
IIIB	1 (1.9)
Grade	
1	2 (3.7)
2	29 (53.7)
3	23 (42.6)
Neo-adjuvant therapy	
Yes	7 (12.7)
No	48 (87.3)

Clinical data, histology, stage, and grade were provided from histopathologic reports submitted by consultant pathologists at University Hospitals of Leicester, Leicester, UK. Stage/Grade information was unavailable for 1 patient sample.

### Optimisation of mIF for evaluating PDE viability

To evaluate BC-PDE viability, mIF staining of FFPE sections using markers for proliferation (Ki67) and apoptosis (cPARP) was performed with a pan-cytokeratin marker (AE1/3) being used to distinguish tumour areas and DAPI for cell nuclei^23,25^. Subsequent digital whole slide scanning and analysis was undertaken as described in the “Methods” Section ^21,23^. Fluorescent signals from individual antibodies were visualised in pseudo-DAB images and AF signals were used to generate pseudo-H&E images (Additional File 4A), which were generated automatically using the Vectra Polaris Image viewing software Phenochart (version 1.0.12). BC-PDEs were segmented into areas of tumour and stroma, while also identifying necrotic areas and background/glass using built-in machine learning algorithms as described in the methods. This was followed by subsequent cell segmentation to identify individual cells, which were phenotyped into Ki67^+ve^, cPARP^+ve^ or DAPI^+ve^ (Additional File 4A). Exemplar images of BC-PDEs with and without chemotherapy treatment (5-fluorouracil, epirubicin and docetaxel; FET) are shown in Additional File 4**B.** Exemplar images of BC-PDE with necrosis are shown in mIF and H&E in Additional File 4C.

### Intrinsic tumour proliferation, apoptosis and necrosis

**Figure 1:**
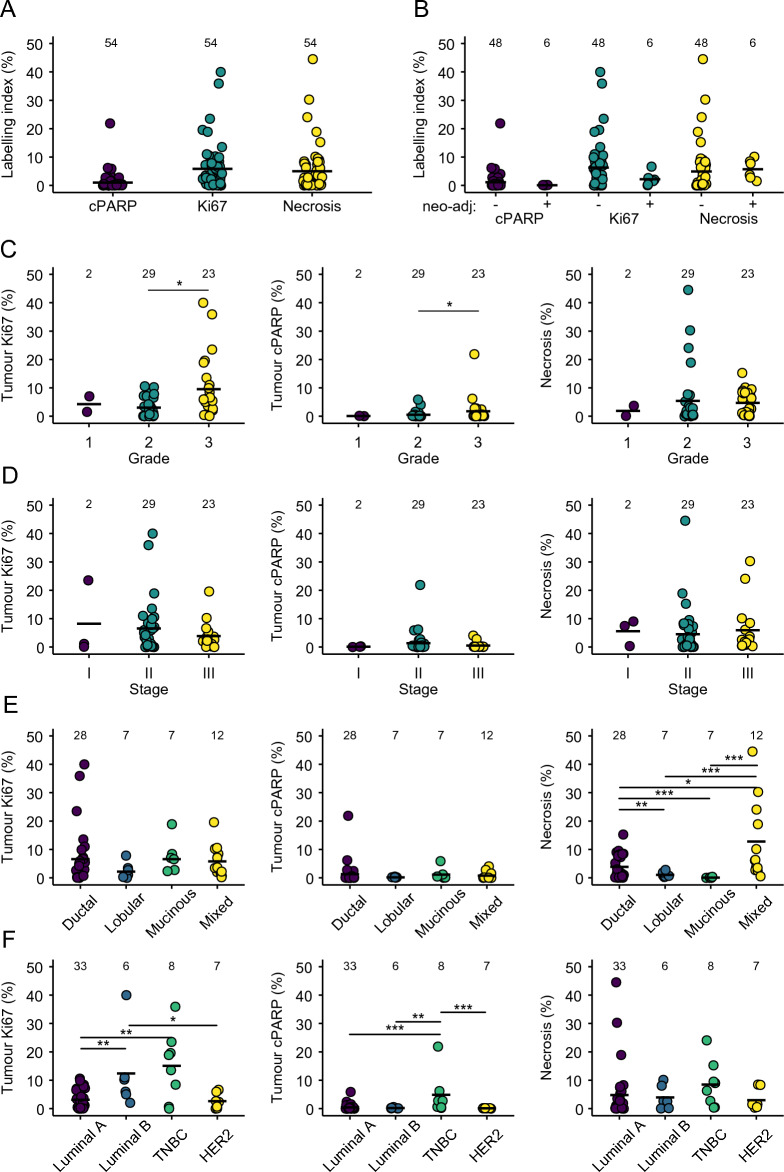
Intrinsic tumour proliferation, apoptosis and necrosis in BC-PDEs. (**A**) % labelling index for Ki67 (proliferation), cPARP (apoptosis) and % necrosis areas in untreated PDEs was derived from 55 patient samples. A single dot represents the median value of respective values for all PDEs derived from a given patient sample. (**B**) Intrinsic levels of tumour proliferation (left), apoptosis (middle) and necrosis (right) in tumour samples from patients with ( +) and without (-) neo-adjuvant therapy. (**C**) Intrinsic levels of tumour proliferation (left), apoptosis (middle) and necrosis (right) according to tumour grade. (**D**) Intrinsic levels of tumour proliferation (left), apoptosis (middle) and necrosis (right) according to tumour stage. (**E**) Intrinsic levels of tumour proliferation (left), apoptosis (middle) and necrosis (right) according to tumour histology. (**F**) Intrinsic levels of tumour proliferation (left), apoptosis (middle) and necrosis (right) according to tumour molecular subtype. Statistics were performed using the Mann–Whitney test, where *p* < 0.05 is indicated by *, *P* < 0.01 is indicated by ** and *p* < 0.001 is indicated by ***. Values displayed above each group represent the number of patient samples in each group.

Using the methodology described above, intrinsic levels of proliferation, apoptosis and necrosis at baseline in tumour areas of untreated BC-PDEs were evaluated in uncultured samples (Fig. 1). BC-PDE proliferation (Ki67^+ve^) ranged from 0–40%, intrinsic tumour apoptosis (cPARP^+ve^) ranged from 0 to 22% and necrosis area ranged from 0 to 45% (Fig. 1A). We investigated the effect of neoadjuvant therapy (NT) on tumour intrinsic proliferation, apoptosis and necrosis, NT did not significantly change these parameters **(**Fig. 1B). Consistent with previous observations, intrinsic tumour proliferation and apoptosis levels were significantly higher in Grade 3 tumours compared to Grade 2 tumours (Fig. 1C)^31,32^. However, there was no significant difference in necrosis between grades. There was no significant difference in the levels of intrinsic proliferation, apoptosis, or necrosis between different tumour stages (Fig. 1D).

Tumour proliferation and apoptosis did not significantly differ between histologic subtypes (Fig. 1E, left and middle panels). Interestingly, however, invasive mucinous carcinomas had significantly less intrinsic necrosis compared to all other histological subtypes whereas tumours of mixed histology and invasive ductal carcinomas had the highest levels of intrinsic necrosis (Fig. 1E, right panel). The reasons for this may be related to fact that tumours classified as “mixed” contained Invasive Ductal Carcinomas (IDCs), and necrosis has been previously reported in ~ 60% of IDC cases^28^. Regarding molecular subtypes, intrinsic proliferation was significantly lower in the Luminal A subgroup compared to Luminal B and Triple Negative Breast Cancers (TNBCs; Fig. 1F left panel), consistent with previous reports^33^. TNBCs also had significantly more intrinsic apoptosis than all other molecular subtypes (Fig. 1F, middle panel) and the highest levels of intrinsic necrosis, although this was not significant (Fig. 1F, right panel). This is consistent with published observations showing that high apoptotic counts are associated with tumours with worse prognosis, as is the case for TNBCs^34^, and that necrotic zones are more prevalent in TNBCs^35^.

### Optimisation of BC-PDE culture

We have previously optimised conditions for the culture of NSCLC- and EC-PDEs and shown maintenance of PDE viability and tumour architecture for up to 48 h^20,21^. With our work in NSCLC-PDEs, we showed that there was a reduction in tissue integrity at 48–72 h, but drug responses correlated with clinical outcomes within the initial 48 h of NSCLC-PDE culture^20^. For the present study, we examined the histopathology of H&E-stained BC-PDE cultures over a time course of up to 92 h and found no evidence for increasing disintegration of tissue integrity up to 68 h (Additional File 5). However, a wider range of tissue disintegration was observed at 92 h of culture (Additional File 5). For this reason, we implemented short-term culture of BC-PDEs, as with the NSCLC-PDEs.

**Figure 2:**
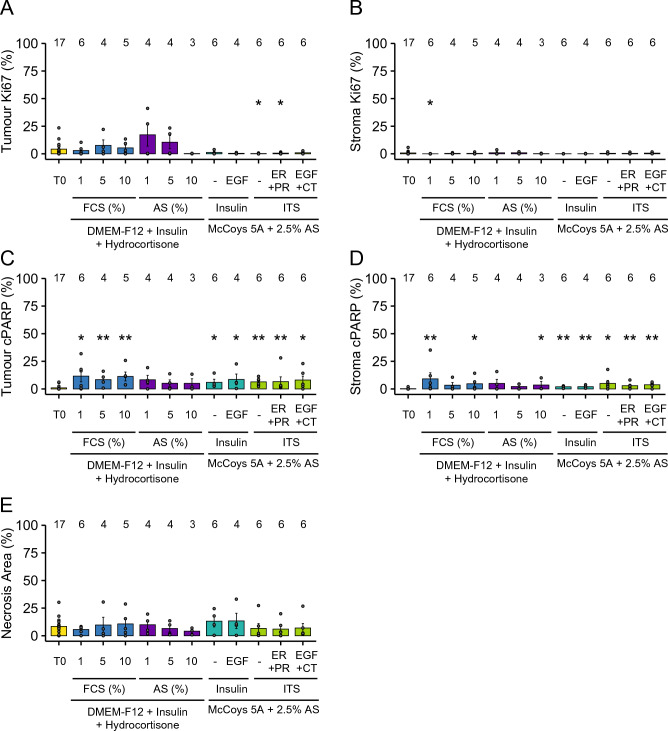
Optimisation of BC-PDE culture. BC-PDEs were cultured in 11 different culture conditions, as detailed in Additional File 2, and compared to uncultured control (T0). (**A**) % Ki67 positivity in tumour (left) or stroma (right) PDEs post-culture compared to uncultured control. (**B**) % cPARP positivity in tumour (left) or stroma (right) PDEs post-culture compared to uncultured control. (**C**) % necrosis area in PDEs post-culture compared to uncultured control. Statistics were performed using the Mann–Whitney test to compare media conditions with T0 reference samples. *p* < 0.05 is shown as * and *p* < 0.01 is shown as **. Values displayed above each group represent the number of patient samples in each group.

To optimise BC-PDE cultures we tested a range of commercially available media and supplements (Additional File 2). Our previous work with EC-PDEs^21^ showed a small but appreciable augmentation in viability with use of autologous serum (AS) and we therefore tested whether AS provides a better alternative to FCS in BC-PDEs. Importantly, previous smaller studies have demonstrated that BC-PDE culture^36^ and culture of normal breast tissue^37^ do not result in altered hormone receptor expression. Therefore, BC-PDEs in this study were assessed only for post-culture levels of Ki67 and cPARP in the tumour and stroma areas, as well as levels of culture-induced necrosis. Due to the small size of some breast cancer samples, it was not possible to culture all patient samples in all media formulations. Therefore, the media conditions compared were acquired from different patient samples, from a mix of molecular and histologic subtypes. There was no significant difference in levels of tumour or stroma proliferation across the various media conditions compared to matched uncultured controls (Fig. 2A, B). However, there were small but significantly higher levels of apoptosis in tumour and stroma areas in BC-PDEs cultured in most media conditions compared to matched uncultured controls (Additional File 2 and Fig. 2C, D). This is consistent with observations for NSCLC-PDEs whereby apoptosis induction following culture was identified as a potential drawback of the PDE methodology although^20^, notably, BC-PDEs underwent considerably lower levels of apoptosis compared to comparative conditions for NSCLC-PDEs (up to 11.6% and 20%, respectively). There was no noticeable variation in apoptosis induction in tumour or stromal areas of PDEs with culture under different media formulations, although the presence of AS appeared to be more beneficial than FCS at augmenting viability (Additional File 2 and Fig. 2C, D), consistent with previous observations for EC-PDEs^21^. There were no significant differences in the levels of culture-induced necrosis in any media formulation compared to matched uncultured controls (Fig. 2E). Overall, these data show that BC-PDE culture does not affect intrinsic proliferation but that there is a slight induction of tumour/stroma apoptosis with culture, with the presence of AS minimising this effect. Thus, 2.5% AS was used as an additive in all future BC-PDE experiments.

### BC-PDE cell death responses to FET chemotherapy

Post-surgical adjuvant therapy for early and locally advanced breast cancer typically utilises one or more of 5 therapeutic options: endocrine therapy, chemotherapy, radiotherapy, bisphosphonate or trastuzumab (see Additional File 1 for details on treatments used for patients recruited to this study). Typical chemotherapeutic regimes for invasive breast cancer contain both an anthracycline and taxane and, in many cases, these are used as part of the FEC-T treatment regime comprising 5-fluorouracil (F), epirubicin (E), docetaxel (T) and cyclophosphamide (C). Of note, cyclophosphamide was omitted from the FET regimen in our study as the cytotoxic activity of cyclophosphamide is derived from hepatic enzymatic bioactivation to the active metabolite 4-hydroxyphosphamide^38^. To evaluate the effect of chemotherapy drugs on BC-PDEs, 46 patient tumours were subjected to PDE derivation and PDEs were cultured for 24 h in the presence or absence of FET. Pharmacologically relevant concentrations of drugs derived from published pharmacokinetic data were utilised (1.15 µM 5-Fluorouracil, 0.1 µM epirubicin, 3 µM docetaxel) for all BC-PDE treatments^39–42^.

In determining cell death responses to FET, we considered fold-change with respect to control as a valid parameter. To determine if this parameter takes into account a possible impact of intrinsic levels of cell death, we assessed the relationship between fold-change cell death with respect to control and percent FET-induced cell death minus control. We found that there were statistically significant positive correlations between these two parameters (Additional File 6). This provided confidence that the fold-change cell death induction parameter mirrors percent cell death changes relative to control and thus we focussed on using this parameter going forward.

**Figure 3:**
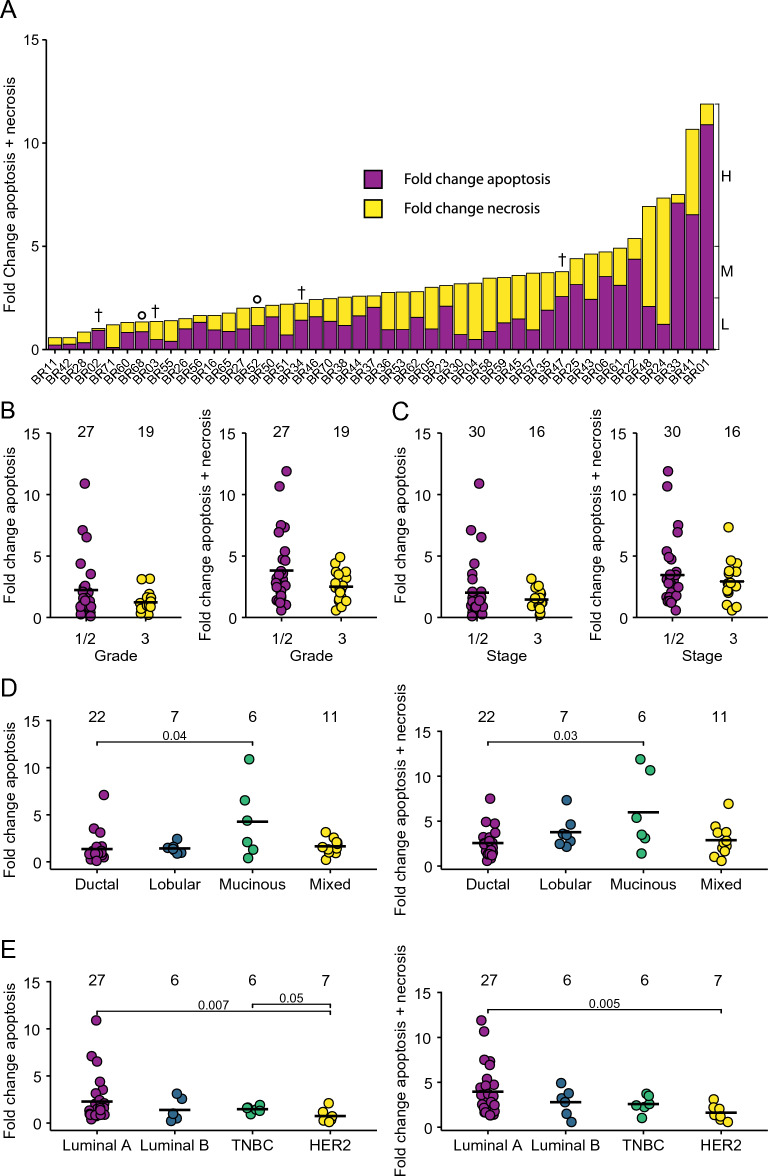
BC-PDE cell death responses to FET chemotherapy. 46 BC-PDEs were treated with FET and a combined value of fold change of drug-induced tumour apoptosis/necrosis compared to vehicle control was calculated for each patient sample. (**A**) BC-PDEs were classified into high (H), medium (M), or low (L) drug-induced apoptosis/necrosis where H =  > 5, M =  > 2.5 and L =  < 2.5-fold change. † indicates PDEs from patients that have subsequently died and ° indicates PDEs from patients that have had a recurrence (Additional File 1). (**B**) Fold change in drug-induced apoptosis (left) or combined apoptosis/necrosis (right) compared to control according to tumour grade. (**C**) Fold change in drug-induced apoptosis (left) or combined apoptosis/necrosis (right) compared to control according to tumour stage. (**D**) Fold change in drug-induced apoptosis (left) or combined apoptosis/necrosis (right) compared to control according to tumour histological subtype. (**E**) Fold change in drug-induced apoptosis (left) or combined apoptosis/necrosis (right) compared to control according to tumour molecular subtype. Statistics were performed using the Mann–Whitney test where *p* < 0.05 are shown. Values displayed above each group represent the number of patient samples in each group.

Fold change levels of tumour apoptosis/necrosis with respect to vehicle control were determined across the 46 samples (Fig. 3A). Samples were classified based on combined levels of drug-induced tumour apoptosis/necrosis into low (< 2.5-fold above control), medium (> 2.5 & < fivefold above control) and high (≥ fivefold above control) (Fig. 3A). The data show a broad range of PDE responses with 43% of cases undergoing a low level of overall cell death, 43% undergoing a medium level and 13% having a high-level induction of cell death in response to FET. Tumours of higher Grade (Fig. 3B) and Stage (Fig. 3C) tended to have lower levels of FET-induced apoptosis or apoptosis/necrosis than lower grade/stage tumours, but none of these comparisons were significant. This is consistent with conflicting reports in the literature linking grade/stage with chemotherapy response in breast cancers^43–45^. However, we found that BC-PDEs from invasive mucinous carcinomas underwent more drug-induced tumour apoptosis and apoptosis/necrosis combined compared to invasive ductal carcinomas (Fig. 3D). These findings are consistent with previous reports showing that invasive mucinous carcinomas respond particularly well to chemotherapy rendering mucin pools almost acellular^46^. In terms of molecular subtypes, HER2-positive tumours underwent significantly less tumour apoptosis and combined tumour apoptosis/necrosis compared to Luminal A and TNBCs (Fig. 3E).

Our mIF analysis at endpoint also allows segregation of drug responses in stromal areas (Additional File 7). As with tumour areas, FET treatment induced a broad range of stromal apoptosis of up to 50-fold higher than vehicle control (Additional File 7A) with the broadest range of apoptosis observed in the Luminal A subtype. Stromal drug-induced apoptosis showed a strong positive correlation with tumour drug-induced apoptosis (Additional File 7B) and combined tumour apoptosis/necrosis (Additional File 7C) showing that FET is indiscriminate in terms of the cell types it targets.

### BC-PDE proliferation responses to FET chemotherapy

**Figure 4:**
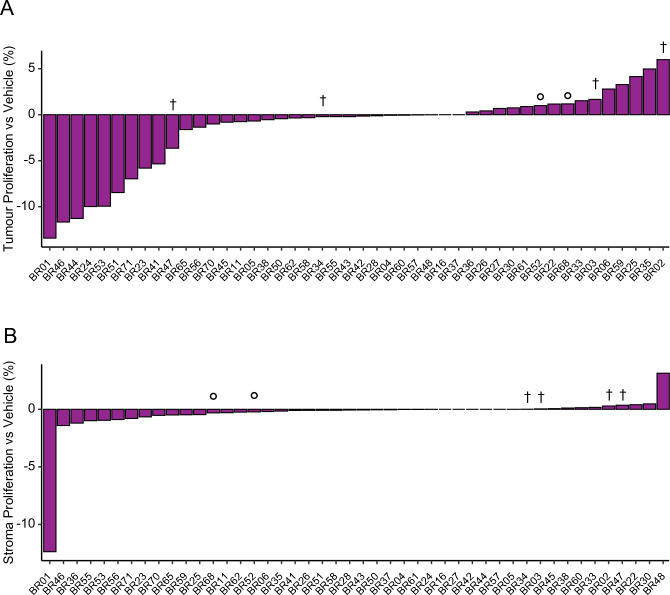
BC-PDE proliferation responses to FET chemotherapy. (**A**)–(**B**) 46 BC-PDEs were treated with FET and percent change in proliferation/Ki67 positivity relative to vehicle control were calculated in tumour and stroma changes, respectively. † indicates PDEs from patients that have subsequently died and ° indicates PDEs from patients that have had a recurrence (Additional File 1).

Docetaxel and epirubicin are known to induce mitotic arrest and inhibit DNA replication, respectively^47,48^. Therefore, we assessed FET-induced changes in proliferation using Ki67 as a marker. This showed that ~ 63% BC-PDEs had lower tumour proliferation (Fig. 4A) and ~ 61% BC-PDEs had lower stroma proliferation (Fig. 4B) compared to vehicle control following FET treatment. Although Ki67 is used clinically as a proliferation marker, it may be slow to respond to short-term drug treatments due to continuous degradation^49^. Therefore, we assessed a second proliferation marker, Geminin. We optimised a mIF staining methodology for Cytokeratin, Ki67 and Geminin (Additional File 8A) and found that there was a significant positive correlation between Geminin^+ve^ and Ki67^+ve^ cells in all PDEs (Additional File 8B). There was also a significant correlation between FET-induced fold change in Ki67 and fold change Geminin (Additional File 8C). Overall, these data show that FET suppresses proliferation in tumour and stromal areas of PDEs as well as inducing cell death changes.

### BC-PDE responses to FET chemotherapy ex vivo correlate with patient outcomes

**Figure 5:**
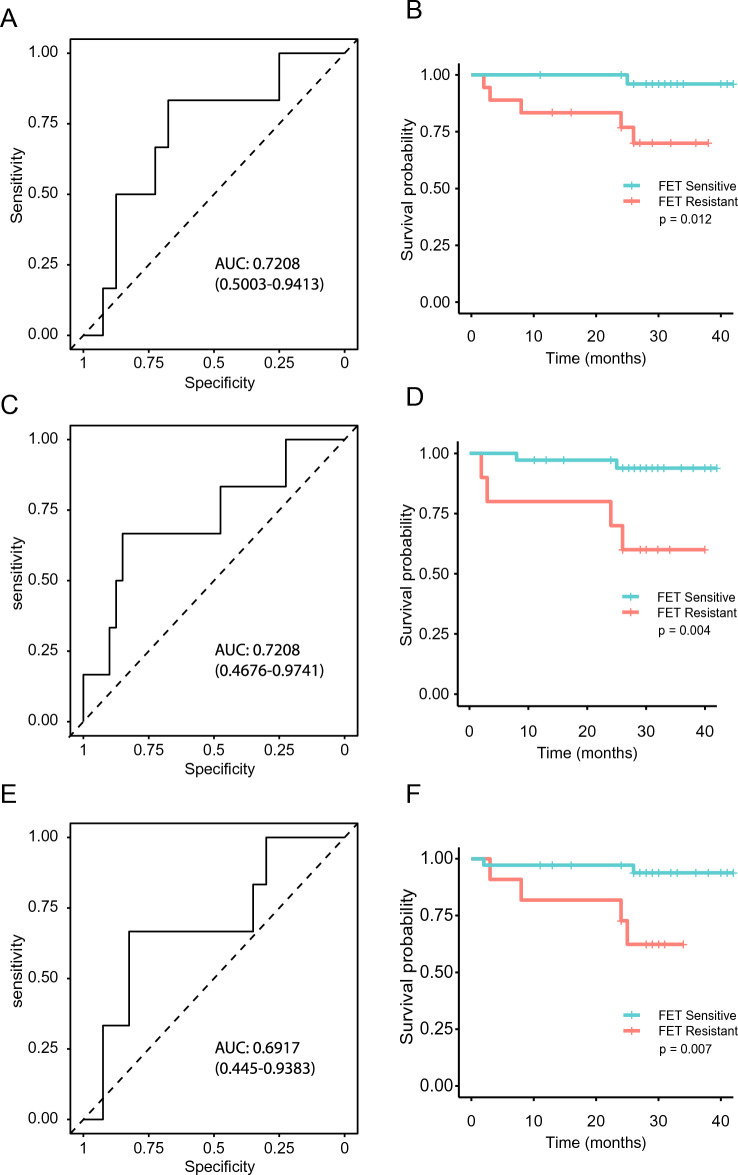
BC-PDE FET responses correlate with clinical outcomes. (**A**) ROC curve used to determine the threshold for resistance/sensitivity to FET treatment using combined fold change tumour apoptosis and necrosis in BC-PDEs. The area under the curve is 0.721 ± 0.105 with an optimal cut off of 2.33 giving 67.5% sensitivity and 83.3% specificity. (**B**) Kaplan–Meier progression free survival for all cases correlated with sensitivity of BC-PDEs to FET with regard to apoptosis and necrosis. (**C**) ROC curve used to determine the threshold for resistance/sensitivity to FET treatment using percent change in FET-treated tumour Ki67 levels relative to vehicle control in BC-PDEs. The area under the curve is 0.721 ± 0.12, with an optimal cut off of 0.933 giving 66.7% sensitivity and 85% specificity. (**D**) Kaplan–Meier progression free survival for all cases correlated with sensitivity of BC-PDEs to FET with regard to change in tumour Ki67. (**E**) ROC curve used to determine the threshold for resistance/sensitivity to FET treatment using percent change in FET-treated stroma Ki67 levels relative to vehicle control in BC-PDEs. The area under the curve is 0.692 ± 0.117, with an optimal cut off of 0.0032 giving 66.7% sensitivity and 82.5% specificity. (**F**) Kaplan–Meier progression free survival for all cases correlated with sensitivity of BC-PDEs to FET with regard to change in stroma Ki67. Statistics for Kaplan–Meier were Mantel-Cox log-rank test, *p* < 0.05 was considered significant.

In the clinic, the patients recruited to this study underwent a range of adjuvant treatments (Additional File 1) and, therefore, for the most part, it was not possible to directly correlate therapeutic response in vivo with ex vivo PDEs response. In addition, due to the success of treatment of BC patients in the UK, most of the patients used for this study have survived > 12 months post-surgery without progression, making comparisons of PDE responses to patient outcomes difficult, unlike the case with NSCLC-PDEs^20^. However, for the 46 samples included in this study, 6 patients had an adverse event, either death or recurrence. A ROC curve was used to determine the threshold for resistance/sensitivity to FET treatment using combined fold change tumour apoptosis and necrosis; this gave an AUC of 0.7208 and identified 2.331837 as the optimal cut off (Fig. 5A). Patient samples were then categorised as either sensitive or resistant to FET and, using the clinical information for corresponding patients, the relationship of FET sensitivity/resistance in PDE culture to patient progression free survival (PFS) was examined (Fig. 5B). This showed a statistically significant relationship (*p* = 0.012), with sensitive cases demonstrating a mean PFS time of 30 months, and resistant cases demonstrating a mean PFS of 23 months. Further to this, we evaluated the relationship with PDE tumour proliferation (Ki67) responses to FET treatment. A ROC curve was generated to determine the threshold for resistance/sensitivity to FET treatment using percentage change in FET-treated tumour Ki67 levels relative to vehicle control in BC-PDEs. This had an AUC of 0.72 (Fig. 5C), showing a statistically significant relationship (*p* = 0.004) with cases defined as sensitive or resistant (Fig. 5D). Interestingly, a ROC curve used to determine the threshold for resistance/sensitivity to FET treatment using percentage change in FET-treated stromal Ki67 relative to vehicle control in BC-PDEs resulted in a ROC curve with an AUC of 0.69 (Fig. 5E) and showed a significant relationship (*p* = 0.007) between patients identified as sensitive/resistant (Fig. 5F). Overall, these data show that BC-PDEs cell death and proliferation responses to FET ex vivo predict short term breast cancer patient outcome.

### BC-PDE responses to Trastuzumab

**Figure 6:**
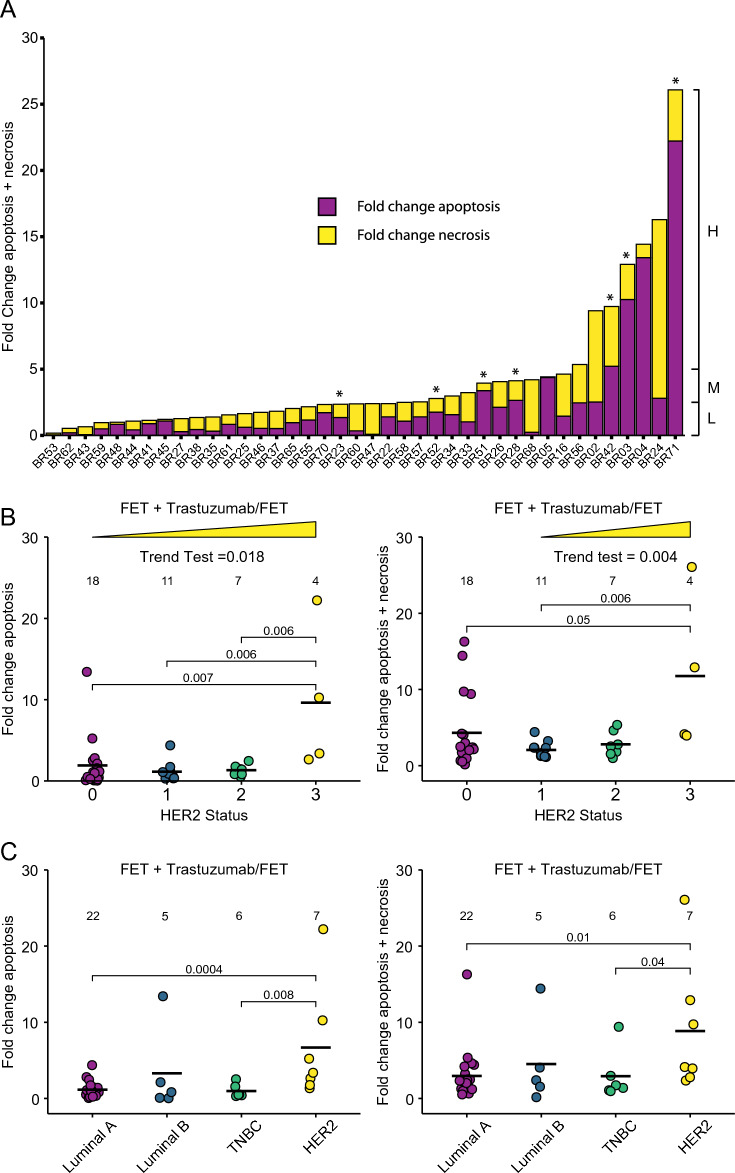
BC-PDE responses to FET + Trastuzumab. 40 BC-PDEs were treated with FET + Trastuzumab. (**A**) Fold change in drug-induced tumour apoptosis/necrosis compared to FET-induced tumour apoptosis/necrosis was determined for each patient sample. BC-PDEs were classified into High (H), Medium (M), and Low (L) drug-induced apoptosis/necrosis where H =  > 5, M =  > 2.5 and L =  < 2.5 fold change combined apoptosis and necrosis . (**B**) Fold change in drug-induced apoptosis (left) or combined apoptosis/necrosis (right) of FET + trastuzumab-treated samples compared to FET alone related to HER2 status. The Jonckheere-Terpstra trend test was performed showing trends for higher HER2 score and higher cell death response. (**C**) Fold change in drug-induced apoptosis (left) or combined apoptosis/necrosis (right) of FET + trastuzumab-treated samples compared to FET only related to molecular subtype. Statistics were performed using the Mann–Whitney test where *p* < 0.05 are shown. Values displayed above each group represent the number of patient samples in each group. * above the stacked bar graph indicates HER2-Enriched molecular subtype.

In previous studies^21,50^, we have demonstrated biological effects in PDEs in response to antibody therapies, suggesting that antibody penetration is possible in this live system. In particular, in malignant melanoma PDEs, we observed the movement of CD8 + cytotoxic T lymphocytes in response to the anti-PD1 antibody Nivolumab^50^, and pembrolizumab-induced death in endometrial cancer PDEs^21^. Indeed, previous studies have shown complete antibody penetration into tissue slices occurs within 20 min^51^. To examine the applicability of the BC-PDE system to the testing of antibody therapies, we derived PDEs from 40 breast cancer samples, and treated them with FET + /− the HER2 therapeutic antibody trastuzumab. As the mechanisms of action for Trastuzumab are still widely controversial, and appear to be dependent on cell-specific factors that differ between individual cancers^52^, there is no single *bona-fide* pharmacodynamic biomarker that can measure Trastuzumab engagement and on-target activity. Therefore, we have relied on Trastuzumab-induced death to assess activity in BC-PDEs, which was calculated by determining fold change apoptosis and combined apoptosis/necrosis relative to FET treatment alone. Using the same classification criteria for drug response as described above, 57.5% underwent low drug-induced tumour apoptosis/necrosis, 25% underwent medium levels of apoptosis/necrosis and 17.5% underwent high levels of drug-induced tumour apoptosis/necrosis (Fig. 6A). Although there were only 4 BC-PDEs with a HER2 score of 3 + , relative to FET treatment alone, there was a significant trend towards higher tumour apoptosis and tumour apoptosis/necrosis dependent on HER2 status (Fig. 6B). Additionally, there were significantly higher levels of tumour apoptosis in BC-PDEs with a HER2 score of 3 + compared to those with HER2 scores of 2 + , 1 + or 0 (Fig. 6B), although there was a high degree of variance amongst these four samples. Due to this high variance, the data were further expressed as percent change in tumour apoptosis and combined tumour apoptosis plus necrosis in FET + Trastzumab compared to FET treatment alone. This showed there was significantly higher tumour apoptosis and combined tumour apoptosis and necrosis (Additional File 9A, B) with a HER2 score of 3 + compared to those with HER2 scores of 0 and 1 + (Additional File 9A) and a score of 1 + (Additional File 9B). Further to this, the high degree of variance observed in Fig. 6B was not present in this analysis. When evaluated across molecular subtypes, 7 patient samples were considered HER2-enriched using the criteria described in the methods. The addition of trastuzumab resulted in significantly higher levels of tumour apoptosis and combined apoptosis/necrosis in HER2 + tumours compared to TNBC and Luminal A tumours (Fig. 6C).

### Maintenance of BC-PDE immune microenvironment

**Figure 7:**
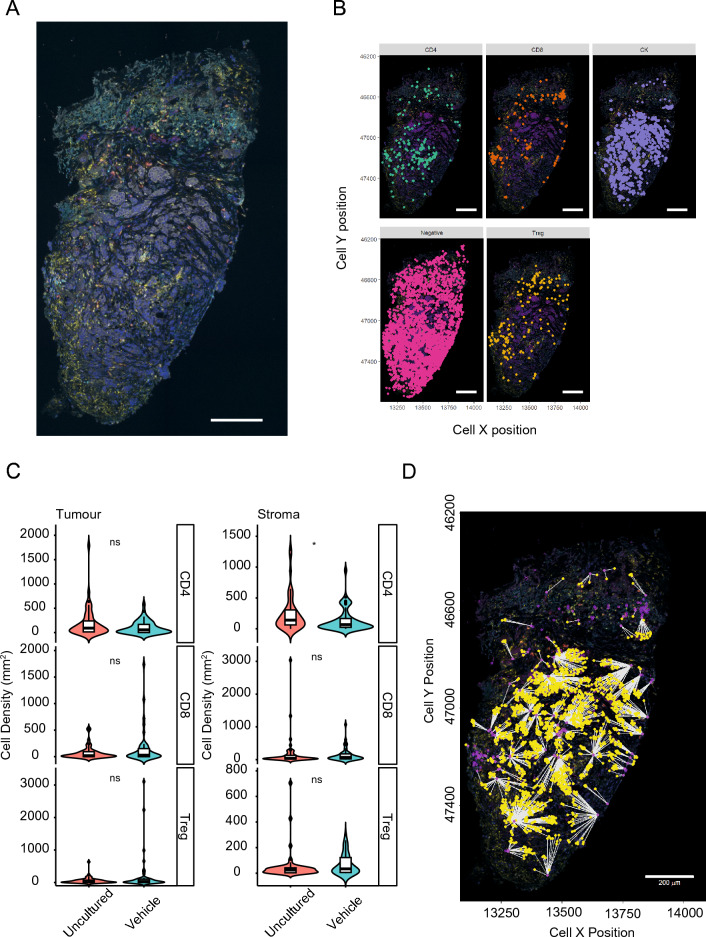
Maintenance of the immune microenvironment in BC-PDEs. Whole slide mIF-stained sections of BC-PDEs were digitised using a Vectra Polaris and analysed in InForm. (A) Representative image of a single PDE stained for CD4, CD8, FOXP3, CK and DAPI. (B) Individual cells were phenotyped into T-Helper (CD4 + ve), T-effector (CD8 + ve), T-regulatory (CD4 + ve FOXP3 + ve), Tumour cells (CK + ve) negative (DAPI + ve). (C) There was no significant difference in cell density/mm2 for T-regulatory, T-effector and T-helper cells in both tumour and stroma in uncultured (48 BC-PDEs) compared to vehicle control (52 BC-PDEs) treated BC-PDEs using Mann–Whitney test, where *p* < 0.05 (indicated by *) was considered significant. (D) Example image of functional inter-cell distance calculation showing Distance from T-effector to CK cells. Scale bars represent 200 µm.

With short-term culture, BC-PDEs retain an intact tumour architecture as based on our H&E assessment (Additional File 5). Since one of our overall goals is the application of the PDE system for evaluating responses to immune checkpoint inhibitors, we further examined the complexity of the BC-PDE immune microenvironment by staining for CD4, CD8, FOXP3 and cytokeratin (Fig. 7A). We identified and phenotyped T-Helper (CD4^+ve^), T-regulatory (CD4^+ve^FOXP3^+ve^) and T-effector (CD8^+ve^) lymphocytes in 100 explants derived from 8 patient samples (Fig. 7B). After 44 h of culture, there was no significant difference in the majority of the lymphocyte subset composition in either the tumour or the stroma (Fig. 7C), although there was a small but significant decrease in stromal CD4 cells. There was also were sufficient numbers of immune cells present in cultured BC-PDEs to undertake functional analysis such as calculating inter-cellular distances between T-effector and tumour cells (Fig. 7D). These data show that BC-PDEs retain a complex stroma and, subject to the ongoing verification of the viability and functionality of these immune cells, gives confidence that BC-PDEs will be applicable to the testing of novel agents such as immunotherapies.

## Discussion

Robust patient-relevant preclinical models for testing novel anti-cancer drugs that have strong predictive value for clinical translation are lacking^50^. Although PDX models can predict drug responses in patients^10,11^ with variable success rates^53^, these models are expensive, have long propagation times and, over time, lose the human tumour microenvironment, thus limiting their clinical utility^50^. In clinical trials, patient-derived organoids were found to be predictive of patient response^54^. However, organoids rely on deconstruction of tumours and 3D culture of dissociated tumour-derived cells, thus destroying the tumour microenvironment, although this can be reconstituted somewhat with addition of heterologous cells. As a consequence of the deconstruction, the amenability of these models for the testing of agents that target the TME is compromised.

In contrast, breast explant methodology has created interest as an alternative preclinical model system. Although breast cancer explants have been utilised for many previous studies as reported in the literature^15–19^, with clinical relevance being demonstrated in some cases^55,56^, many of these other methodologies result in loss of tumour architecture over time, which compromises the ability to monitor changes in the tumour microenvironment in response to drug treatment. It is for this reason that we have optimised the short-term culture of intact breast cancer PDEs. Our data show that tumour architecture is maintained for up to 72 h of culture (Fig. 2 and Additional File 5) and, importantly, the immune microenvironment is preserved (Fig. 7), which is highly encouraging for the testing of agents such as immune checkpoint inhibitors that target immune cells of the TME.

The culture method we have employed resulted in minimal changes in culture-induced tumour and stroma proliferation, apoptosis, and necrosis (Fig. 2). While culture did increase tumour apoptosis by up to 10%, this was reduced by inclusion of autologous serum, consistent with results previously obtained for EC- and HNSCC-PDEs^21,22^. It is reasoned that autologous serum contains a patient/tumour specific milieu of growth-factors more closely resembling in vivo conditions^22^ that together contribute to increased PDE integrity. Overall, although short term culture of explants has limitations including the fact that acquired drug resistance cannot be assessed, the retention of an intact tumour microenvironment is a significant advantage over other culture methods.

In this study, we have also introduced a technical advancement of implementing mIF and whole-slide scanning coupled to digital pathology solutions to increase the accuracy and automation of biomarker expression in response to drug treatment in both the tumour and stroma regions of BC-PDEs. As we have shown, this presents a significant improvement compared to more traditional methods of histological methods of DAB immunostaining and MTT incorporation analysis, as multiple biomarkers can be assessed simultaneously in different regions of the tumour and quantitation of the staining becomes more amendable.

Our data show that BC-PDE responses to drug treatment reflect clinical expectations in a number of ways. Firstly, as in the clinic, cell death responses to FET treatment in PDEs is dependent on histological subtype: mucinous carcinomas, which typically have a more favourable prognosis clinically^57^, undergo significantly more tumour apoptosis/necrosis in response to FET compared to invasive ductal carcinomas while, in terms of molecular subtypes, HER2-positive tumours underwent significantly less apoptosis/necrosis compared to Luminal A and TNBCs**.** Secondly, ROC and Kaplan–Meier analysis demonstrates a significant relationship between BC-PDEs proliferation and cell death responses to FET and PFS in patients. Thirdly, the addition of the HER2 antagonist trastuzumab to FET treatment resulted in a significant increase in tumour cell death that correlated with HER2 status, and HER2-enriched molecular subtypes also underwent significantly higher cell death responses compared to both TNBC and luminal A subgroups. All of these data point to a strong link between drug responses observed preclinically in PDEs and those that occur clinically^58,59^. The data also show that the platform can be utilised for the testing of chemotherapeutics, as in the case of FET, as well as targeted and antibody-directed therapies, as in the case of trastuzumab.

## Conclusions

Overall, our data show that drug responses in the BC-PDE platform are reflective of patient outcomes when assessing responses to both standard of care chemotherapy drugs as well as antibody-directed and targeted therapies. Our data show that BC-PDEs retain the tumour immune microenvironment and are accessible to therapeutic antibodies. This is particularly encouraging for the application of this platform to the preclinical testing of immune checkpoint inhibitors, for which an appropriate preclinical testing platform is urgently sought.

### Supplementary Information

Supplementary Information 1.

Supplementary Information 2.

Supplementary Information 3.

Supplementary Information 4.

Supplementary Information 5.

Supplementary Information 6.

Supplementary Information 7.

Supplementary Information 8.

Supplementary Information 9.

## Data Availability

The datasets generated and used during the current study are available from the corresponding author on reasonable request.

## Abbreviations

PDE: Patient-derived explant
BC-PDE: Breast cancer patient-derived explant
FET: 5-Fluoruracil, epirubicin, docetaxel
TNBC: Triple negative breast cancer
TSR: Tumour stroma ratio
HER2: Human epidermal growth factor receptor 2
NSCLC: Non-small cell lung cancer
HNSCC: Head and neck squamous cell carcinoma
EC: Endometrial cancer
mIF: Multiplexed immune-fluorescence
AS: Autologous serum
FCS: Foetal calf serum
IDC: Invasive ductal carcinoma
