# Supplementary material for: An optimised patient-derived explant platform for breast cancer reflects clinical responses to chemotherapy and antibody-directed therapy
Source: Sci Rep. 2024 Jun 4;14:12833. doi: 10.1038/s41598-024-63170-0 (PMC11150370; doi:10.1038/s41598-024-63170-0)
Supplement: Supplementary file 1 — Supplementary Information 1. [file 41598_2024_63170_MOESM1_ESM.docx]

**An optimised patient-derived explant platform for breast cancer reflects clinical responses to chemotherapy and antibody-directed therapy**

Constantinos Demetriou^1^, Naila Abid^1^, Michael Butterworth^1^, Larissa Lezina^1^, Pavandeep Sandhu^1^, Lynne Howells^1^, Ian R Powley^1^, James Howard Pringle^1^, Zahirah Sidat^2^, Omar Qassid^1,3^, Dave Purnell^3^, Monika Kaushik^4^, Kaitlin Duckworth^4^, Helen Hartshorn^4^, Anne Thomas^1^, Jacqui A Shaw^1^, Marion MacFarlane^5,6^*, Catrin Pritchard^1^*, Gareth J Miles^1^*

*Additional File 1. Summary of patient and tumour characteristics*

CT = Chemotherapy (CT1; Docetaxel, CT2; Epirubicin + Cyclophosphamide, CT3; Fluorouracil + Epirubicin + Cyclophosphamide, CT4; Paclitaxel, CT5; Denosumab)), ET = Endocrine therapy (ET1; Letrozole, ET2; Tamoxifen, ET3; Anastrozole, ET4; Zoladex) RT = Radiotherapy, BT = Bisphosphonate therapy (BT1; Zoledronic acid, BT2; Ibandronic acid, BT3; Alendronic acid), P = Pertuzumab, H = Herceptin, TNBC = Triple negative breast cancer. * = data unavailable. Where treatments are separated by / this indicates treatments have been switched. In Months after surgery column, where two values are provided and separated by a / the first is months to detected recurrence, the second is months elapsed to survival/death. Where HER-Enriched subtype has a *, these samples were identified as HER2 amplified by Whole Exome Sequencing

| Patient ID | Age | Histology | ER Score | HER2 Score | Subtype | Stage | Grade | Neo-Adjuvant Therapy | Adjuvant Therapy | Survival Status | Months after Surgery (date) |
| --- | --- | --- | --- | --- | --- | --- | --- | --- | --- | --- | --- |
| BR01 | 43 | Mucinous Carcinoma | 8 | 0 | Luminal A | IIA | 2 | None | ET2 | Alive | 42 |
| BR02 | 72 | Ductal-Lobular Carcinoma | 0 | 0 | TNBC | IIIA | 2 | None | CT1 + BT1 | Deceased | 24 |
| BR03 | 73 | Ductal Carcinoma | 0 | 3 | HER2-Enriched | IIB | 3 | None | None | Deceased | 3 |
| BR04 | 65 | Ductal Carcinoma | 8 | 0 | Luminal B | IIA | 3 | None | ET1 + RT | Alive | 41 |
| BR05 | 85 | Ductal Carcinoma | 8 | 1 | Luminal A | IB | 2 | None | ET1 + RT | Alive | 40 |
| BR06 | 54 | Ductal Carcinoma | 8 | 1 | Luminal A | IIB | 2 | None | RT + ET3 | Alive | 40 |
| BR08 | 56 | Ductal Carcinoma | 8 | 2 | Luminal A | IIA | 3 | None | ET1 | Alive | 40 |
| BR09 | 63 | Mucinous Carcinoma | 8 | 0 | Luminal A | IIA | 2 | None | ET1 | Alive | 40 |
| BR11 | 72 | Ductal-Lobular Carcinoma | 8 | 1 | Luminal B | IIIA | 3 | ET1 | CT2 + BT1 + BT2 | Alive | 38 |
| BR12 | 77 | Ductal Carcinoma | 3 | 0 | TNBC | IB | 3 | None | ET1/2 + RT | Alive | 38 |
| BR13 | 70 | Ductal Carcinoma | 0 | 0 | TNBC | IIA | 3 | None | CT3 + CT1 | Deceased | 14 |
| BR14 | 68 | Ductal-Lobular Carcinoma | 8 | 1 | Luminal A | IIA | 2 | None | RT + ET3 | Alive | 37 |
| BR16 | 68 | Ductal Carcinoma | 8 | 2 | Luminal A | IIB | 3 | None | CT2 + BT1/2 + CT1 + RT + ET3 | Alive | 36 |
| BR20 | 57 | Ductal Carcinoma | 8 | 1 | Luminal A | IIB | 2 | None | CT2 + CT1 + RT + BT2/1 + ET1 | Alive | 35 |
| BR21 | 40 | Ductal Carcinoma | 7 | 1 | Luminal A | IIA | 2 | None | ET2 | Alive (Recurrence 24 months) | 24/34 |
| BR22 | 83 | Mucinous Carcinoma | 8 | 0 | Luminal A | IIA | 2 | None | RT + ET1/3 + BT3 | Alive | 34 |
| BR23 | 85 | Mucinous Carcinoma | 8 | 1 | HER2-Enriched* | IIA | 2 | None | ET2 | Alive | 33 |
| BR24 | 58 | Lobular Carcinoma | 7 | 0 | Luminal A | IIIA | 2 | None | CT2 + RT + ET1/3 + BT2 | Alive | 33 |
| BR25 | 49 | Ductal-Lobular Carcinoma | 8 | 1 | Luminal A | IIIA | 3 | None | CT2 + RT + ET3 + ET4 | Alive | 32 |
| BR26 | 48 | Ductal Carcinoma | 7 | 0 | Luminal B | IIA | 3 | None | CT3 + BT1/2 + ET2/3 | Alive | 32 |
| BR27 | 84 | Ductal-Lobular Carcinoma | 8 | 1 | Luminal A | IIIA | 1 | ET2 | ET2 + RT | Alive | 32 |
| BR28 | 77 | Ductal Carcinoma | 8 | 3 | HER2-Enriched | IIIA | 3 | ET1 | CT4 + H + ET1 + RT | Alive | 32 |
| BR30 | 67 | Ductal Carcinoma | 8 | 0 | Luminal A | IIA | 2 | ET1 | RT + ET1/2 | Alive | 31 |
| BR33 | 61 | Ductal Carcinoma | 8 | 1 | Luminal A | IIB | 2 | None | CT3 + RT + ET1 + BT2 | Alive | 30 |
| BR34 | 76 | Squamous Differentiation-Metaplastic Carcinoma | 0 | 0 | TNBC | IIA | 3 | None | * | Deceased | 8 |
| BR35 | 70 | Ductal-Metaplastic Carcinoma | 0 | 1 | TNBC | IIIA | 3 | None | RT | Alive | 29 |
| BR36 | 42 | Lobular Carcinoma | 7 | 1 | Luminal A | IIIA | 2 | None | CT2 + CT4 + RT + ET2 + ET4 | Alive | 30 |
| BR37 | 85 | Ductal-Lobular Carcinoma | 8 | 2 | Luminal A | IIIB | 2 | None | RT + ET1 + BT3 | Alive | 30 |
| BR38 | 48 | Ductal Carcinoma | 8 | 1 | Luminal A | IIIA | 3 | None | CT3 + CT1 + RT + ET2 | Alive | 29 |
| BR41 | 85 | Mucinous Carcinoma | 8 | 1 | Luminal A | IIA | 1 | None | ET2 | Alive | 28 |
| BR42 | 69 | Ductal Carcinoma | 8 | negative | HER2-Enriched* | IA | 2 | None | RT + ET1 | Alive | 29 |
| BR43 | 57 | Ductal-Lobular Carcinoma | 8 | 0 | Luminal A | IIIA | 2 | None | CT2 + BT1 + ET3 + RT + BT2 | Alive | 28 |
| BR44 | 73 | Ductal Carcinoma | 0 | 0 | TNBC | IIA | 3 | None | CT2 | Alive | 28 |
| BR45 | 64 | Lobular Carcinoma | 8 | 1 | Luminal A | IIIA | 2 | None | CT3 + CT1 + ET1 + BT2 + RT | Alive | 28 |
| BR46 | 50 | Ductal Carcinoma | 3 | 0 | TNBC | IIA | 3 | None | CT3 + CT1 + RT + BT2 | Alive | 28 |
| BR47 | 84 | Ductal-Lobular Carcinoma | 8 | 0 | Luminal B | IIIA | 2 | None | RT + ET1 + BT2 | Deceased | 25 |
| BR48 | 79 | Ductal-Lobular Carcinoma | 8 | 2 | Luminal A | IIA | 2 | None | RT + ET1 + BT2 | Alive | 28 |
| BR50 | 76 | Lobular Carcinoma | 8 | 1 | Luminal A | IIIA | 2 | None | ET1 + RT | Alive | 27 |
| BR51 | 76 | Ductal Carcinoma | 8 | 3 | HER2-Enriched | IIIA | 3 | ET1 | CT2 + CT1 + P + H + RT + ET3 | Alive | 27 |
| BR52 | 35 | Ductal Carcinoma | 8 | 2 | HER2-Enriched* | IIA | 3 | None | CT3 + RT + ET2 | Alive (Recurrence 26 months) | 26/27 |
| BR53 | 56 | Ductal Carcinoma | 8 | 0 | Luminal B | IIA | 3 | None | CT3 + RT + ET1 + BT2 | Alive | 26 |
| BR55 | 49 | Mucinous Carcinoma | 8 | 0 | Luminal A | IIB | 2 | None | RT + ET3/2 | Alive | 26 |
| BR56 | 87 | Ductal-Lobular Carcinoma | 8 | 2 | Luminal A | IIB | 2 | None | ET1 | Alive | 26 |
| BR57 | 47 | Ductal Carcinoma | 8 | 2 | Luminal A | IIB | 3 | None | RT | Alive | 26 |
| BR58 | 56 | Lobular Carcinoma | 8 | 0 | Luminal A | IIB | 2 | None | RT + ET1 + BT2 | Alive | 26 |
| BR59 | 77 | Mucinous Carcinoma | 0 | 0 | TNBC | IIA | 3 | None | RT + BT2 | Alive | 26 |
| BR60 | 66 | Ductal Carcinoma | 8 | 1 | Luminal A | IIB | 2 | None | ET1 | Alive | 24 |
| BR61 | 71 | Ductal Carcinoma | 8 | 2 | Luminal B | IIA | 3 | None | CT3 + RT + ET3 + BT2 | Alive | 24 |
| BR62 | 59 | Ductal-Lobular Carcinoma | 8 | 0 | Luminal A | IIIA | 2 | None | CT3 + CT1 + ET3 + RT + BT2 | Alive | 24 |
| BR64 | 51 | Ductal Carcinoma | 8 | 1 | Luminal A | IIA | 3 | None | CT2 + CT1 | Alive | 16 |
| BR65 | 54 | Ductal Carcinoma | 8 | negative | Luminal A | IIB | 2 | None | CT2 + CT1 | Alive | 16 |
| BR68 | 49 | Ductal Carcinoma | 8 | negative | Luminal A | IIA | 3 | None | CT5 + ET1 + BT2 | Alive (Recurrence 2 months) | 2/14 |
| BR70 | 58 | Lobular Carcinoma | 8 | 1 | Luminal A | IIA | 2 | ET1 | RT +ET1 | Alive | 11 |
| BR71 | 78 | Ductal Carcinoma | 8 | 3 | HER2-Enriched | IIA | 2 | None | RT +ET1 | Alive | 13 |
| BR88 | - | Lobular carcinoma | 8 | 0 | Luminal A | - | - | ET1 | - | Alive | 1 |
