# Supplementary material for: An optimised patient-derived explant platform for breast cancer reflects clinical responses to chemotherapy and antibody-directed therapy
Source: Sci Rep. 2024 Jun 4;14:12833. doi: 10.1038/s41598-024-63170-0 (PMC11150370; doi:10.1038/s41598-024-63170-0)
Supplement: Supplementary file 2 — Supplementary Information 2. [file 41598_2024_63170_MOESM2_ESM.docx]

**An optimised patient-derived explant platform for breast cancer reflects clinical responses to chemotherapy and antibody-directed therapy**

Constantinos Demetriou^1^, Naila Abid^1^, Michael Butterworth^1^, Larissa Lezina^1^, Pavandeep Sandhu^1^, Lynne Howells^1^, Ian R Powley^1^, James Howard Pringle^1^, Zahirah Sidat^2^, Omar Qassid^1,3^, Dave Purnell^3^, Monika Kaushik^4^, Kaitlin Duckworth^4^, Helen Hartshorn^4^, Anne Thomas^1^, Jacqui A Shaw^1^, Marion MacFarlane^5,6^*, Catrin Pritchard^1^*, Gareth J Miles^1^*

## *Additional File 2. Summary of BC-PDE apoptosis, proliferation and necrosis levels following culture under different conditions.*

Tumour and Stroma proliferation (Ki67), apoptosis (cPARP) and necrotic indexes of BC-PDEs under all culture conditions are shown. Data expressed as mean (± SEM). SEM = standard error of the mean, FCS = Foetal Calf Serum, AS = Autologous Serum, AS = Autologous Serum, EGF = Epidermal Growth Factor. N = number of patient samples used, where each patient sample was represented by 6-9 PDEs. T0 = uncultured control

| Media | Serum | Supplements | n | Tumour Proliferation | Stroma Proliferation | Tumour Apoptosis | Stroma Apoptosis | Necrosis |
| --- | --- | --- | --- | --- | --- | --- | --- | --- |
|  | | |  | % mean (± SEM) | | | | |
| T0 | | | 17 | 4.2 ± 1.33 | 0.8 ± 0.4 | 0.9 ± 0.5 | 0.2 ± 0.1 | 8.4 ± 1.8 |
| DMEM-F12 | 1% FCS | 1 µg/mL Insulin + 5 µg/mL hydrocortisone | 6 | 3 ± 1.6 | 0 ± 0 | 11.6 ± 5.2 | 9.1 ± 5.5 | 5.6 ± 1.3 |
|  | 5% FCS |  | 4 | 7.6 ± 5 | 0.4 ± 0.3 | 8.5 ± 3.2 | 3.4 ± 2.4 | 9.7 ± 7.1 |
|  | 10% FCS |  | 5 | 5.4 ± 2.6 | 0.4 ± 0.3 | 11.3 ± 4 | 4.7 ± 2.6 | 10.7 ± 5.2 |
|  | 1% AS |  | 4 | 17.2 ± 10.2 | 0.9 ± 0.9 | 8.3 ± 4 | 5 ± 3.7 | 9.9 ± 4 |
|  | 5% AS |  | 4 | 10.5 ± 6 | 0.9 ± 0.5 | 5.1 ± 3.1 | 2.2 ± 1.3 | 6.5 ± 3.1 |
|  | 10% AS |  | 3 | 0.3 ± 0.1 | 0.2 ± 0.2 | 5 ± 4.2 | 3.5 ± 3.2 | 4.1 ± 1.8 |
| McCoy’s 5A | 2.5% AS | 5 ng/mL Insulin + 1X sodium pyruvate | 6 | 1.5 ± 0.6 | 0.2 ± 0.1 | 7.2 ± 2.2 | 4.8 ± 1.9 | 13.2 ± 5.2 |
|  |  | 5 ng/mL Insulin + 1X sodium pyruvate + 20 ng/mL EGF | 4 | 0.4 ± 0.3 | 0.1 ± 0.1 | 8.6 ± 4.9 | 2 ± 0.7 | 13.4 ± 7 |
|  |  | 1X ITS + 1X sodium pyruvate | 6 | 0.2 ± 0.1 | 0.5 ± 0.3 | 6.4 ± 1.4 | 5 ± 2.7 | 6.6 ± 4.3 |
|  |  | 1X ITS + 1X sodium pyruvate + 10 nM Estradiol + 10 nM Progesterone | 6 | 0.6 ± 0.3 | 0.4 ± 0.2 | 6.6 ± 4.3 | 2.9 ± 1.1 | 6 ± 3.1 |
|  |  | 1X ITS + 1X sodium pyruvate + 20 ng/mL EGF + 100 ng/mL Cholera Toxin | 6 | 1 ± 0.4 | 0.7 ± 0.3 | 8 ± 3.6 | 3.8 ± 0.9 | 7 ± 4 |
