# Supplementary material for: An optimised patient-derived explant platform for breast cancer reflects clinical responses to chemotherapy and antibody-directed therapy
Source: Sci Rep. 2024 Jun 4;14:12833. doi: 10.1038/s41598-024-63170-0 (PMC11150370; doi:10.1038/s41598-024-63170-0)
Supplement: Supplementary file 3 — Supplementary Information 3. [file 41598_2024_63170_MOESM3_ESM.docx]

| **Antibody** | **1° Ab Dilution** | **Incubation time** | **HIER** | **Fluor** |
| --- | --- | --- | --- | --- |
| *Viability Panel* | | | | |
| Ki67 (DAKO-M7240) | 1:1000 | 30 min, RT | TE | Opal-520 |
| CK AE1/AE3 (DAKO-M3515) | 1:400 | 30 min, RT | TE | Opal-570 |
| Cleaved PARP (Abcam-Ab32064) | 1:2000 | 30 min, RT | TE | Opal-690 |
| *Geminin Panel* | | | | |
| Ki67 (DAKO-M7240) | 1:1000 | 30 min, RT | TE | Opal-520 |
| Geminin (Abcam-Ab195047) | 1:500 | Overnight, 4°C | TE | Opal-690 |
| CK AE1/AE3 (DAKO-M3515) | 1:400 | 30 min, RT | TE | Opal-570 |
| *Immune cell panel* | | | | |
| CD8 (DAKO-M7103) | 1:100 | 30 min, RT | TE | Opal-480 |
| Fox-P3 (Invitrogen-14-4777-82) | 1:100 | 30 min, RT | TE | Opal-520 |
| CD4 (DAKO-M7310) | 1:80 | 30 min, RT | TE | Opal-570 |
| CK AE1/AE3 (DAKO-M3515) | 1:400 | 30 min, RT | TE | Opal 690 |

**An optimised patient-derived explant platform for breast cancer reflects clinical responses to chemotherapy and antibody-directed therapy**

Constantinos Demetriou^1^, Naila Abid^1^, Michael Butterworth^1^, Larissa Lezina^1^, Pavandeep Sandhu^1^, Lynne Howells^1^, Ian R Powley^1^, James Howard Pringle^1^, Zahirah Sidat^2^, Omar Qassid^1,3^, Dave Purnell^3^, Monika Kaushik^4^, Kaitlin Duckworth^4^, Helen Hartshorn^4^, Anne Thomas^1^, Jacqui A Shaw^1^, Marion MacFarlane^5,6^*, Catrin Pritchard^1^*, Gareth J Miles^1^*

*Additional File 3. Antibody-Fluorophore pairs, and staining conditions for 3 mIF panels used in the study*.

All Opal Reagents were purchased from Akoya Biosciences. RT = room temperature. TE = Tris-EDTA buffer, pH9.
