# Supplementary material for: An optimised patient-derived explant platform for breast cancer reflects clinical responses to chemotherapy and antibody-directed therapy
Source: Sci Rep. 2024 Jun 4;14:12833. doi: 10.1038/s41598-024-63170-0 (PMC11150370; doi:10.1038/s41598-024-63170-0)

An optimised patient-derived explant platform for breast cancer reflects clinical responses to chemotherapy and antibody-directed therapy

Constantinos Demetriou[1], Naila Abid[1], Michael Butterworth[1], Larissa Lezina[1], Pavandeep Sandhu[1], Lynne Howells[1], Ian R Powley[1], James Howard Pringle[1], Zahirah Sidat[2], Omar Qassid[1,3], Dave Purnell[3], Monika Kaushik[4], Kaitlin Duckworth[4], Helen Hartshorn[4], Anne Thomas[1], Jacqui A Shaw[1], Marion MacFarlane[5,6]*, Catrin Pritchard[1]*, Gareth J Miles[1]*

Additional File 4. Optimisation of multiplexed Immuno-Fluorescence

Whole slide mIF-stained FFPE sections of BC-PDEs were digitised using a Vectra Polaris and analysed in InForm. **A**, Captured AF, Ki67, CK and cPARP staining was used to generate pseudo-H&E and -DAB images. InForm was then trained to segment tissue into tumour (yellow), stroma (green), necrosis (red) and background/glass (blue). Next, cells were segmented and phenotyped into Ki67 (green), cPARP (Red) and DAPI (blue). **B,** Representative examples of vehicle- and FET-treated BC-PDEs showing high Ki67 staining in vehicle samples and low Ki67 staining in FET-treated samples as well as high cPARP staining in FET-treated samples, with low staining in vehicle samples. Scale bars = 50 µm. **C,** Representative examples of necrosis shown in mIF, with CK+ve cPARP+ve DAPI-ve, and as a H&E. Scale bars = 20 µm.

Additional File 4

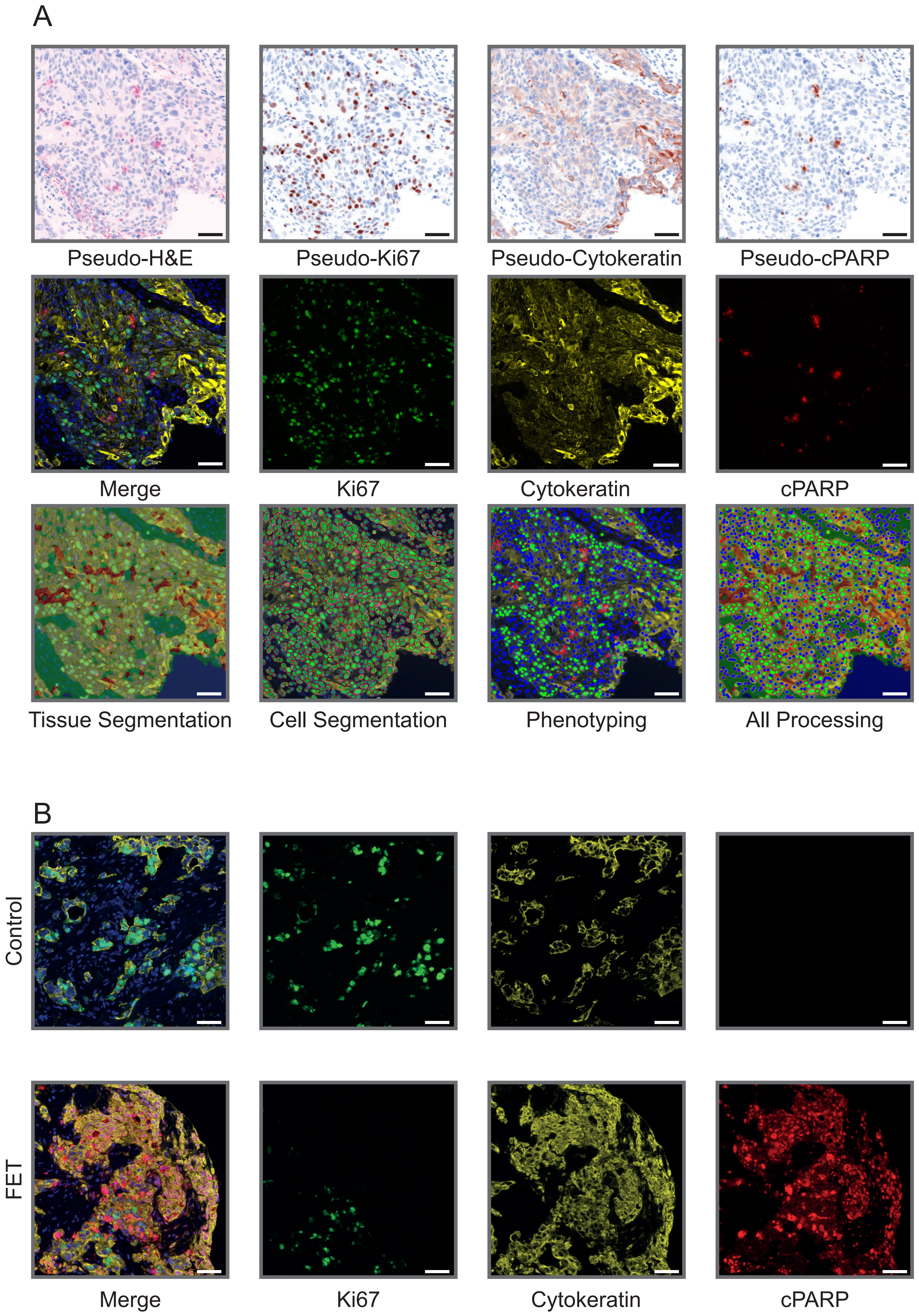

Additional File 4

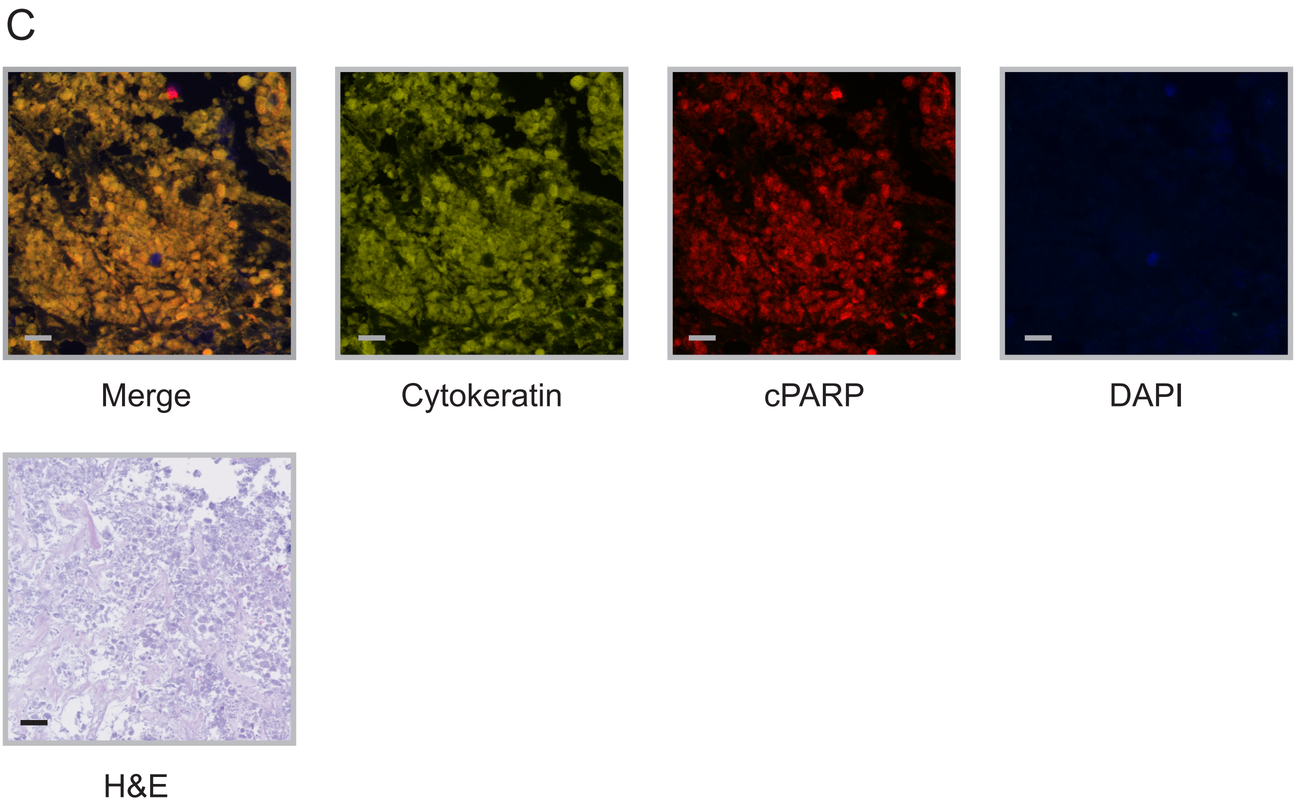

Supplement: Supplementary file 4 — Supplementary Information 4. [file 41598_2024_63170_MOESM4_ESM.pdf]
