# Supplementary material for: An optimised patient-derived explant platform for breast cancer reflects clinical responses to chemotherapy and antibody-directed therapy
Source: Sci Rep. 2024 Jun 4;14:12833. doi: 10.1038/s41598-024-63170-0 (PMC11150370; doi:10.1038/s41598-024-63170-0)

# An optimised patient-derived explant platform for breast cancer reflects clinical responses to chemotherapy and antibody-directed therapy

Constantinos Demetriou[1], Naila Abid[1], Michael Butterworth[1], Larissa Lezina[1], Pavandeep Sandhu[1], Lynne Howells[1], Ian R Powley[1], James Howard Pringle[1], Zahirah Sidat[2], Omar Qassid[1,3], Dave Purnell[3], Monika Kaushik[4], Kaitlin Duckworth[4], Helen Hartshorn[4], Anne Thomas[1], Jacqui A Shaw[1], Marion MacFarlane[5,6]*, Catrin Pritchard[1]*, Gareth J Miles[1]*

Additional File 5. Time course of BC-PDE culture

BC-PDEs were cultured for 20 hours (O/N) to recover from processing and then cultured for a further 24, 48 and 72 h. total culture times were 44, 68 and 92 h, respectively. T0 = 0 hr baseline control. Scale bars in H&E's represent 100 µm and arrows show groups of invasive tumour cells.

# Additional File 5

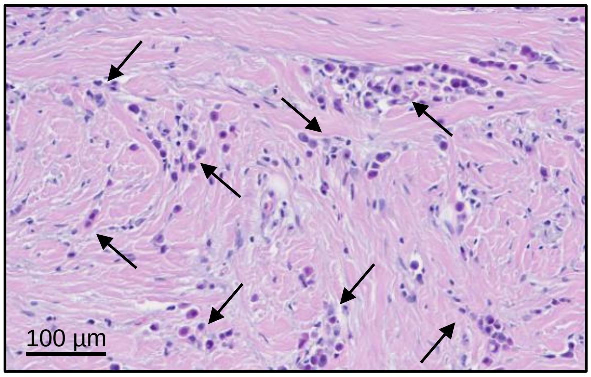


T0

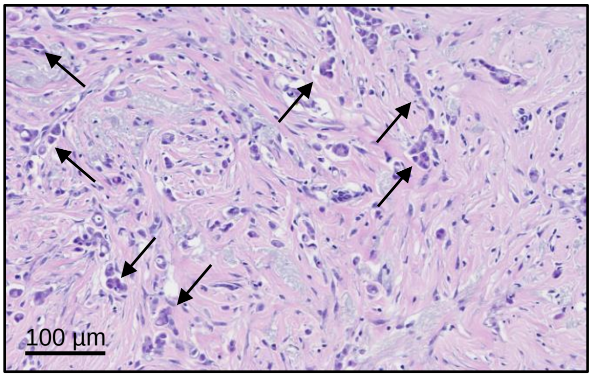


20 h O/N + 24 h

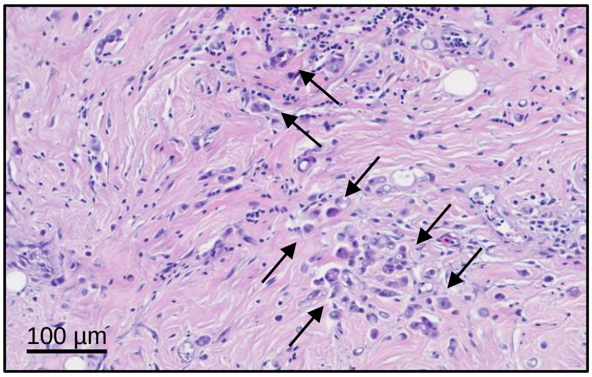


20 h O/N + 48 h

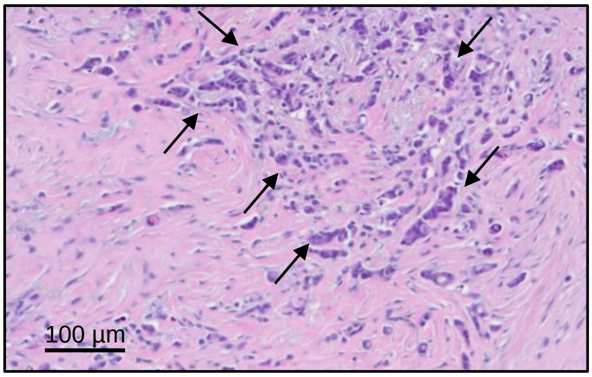


20 h O/N + 72 h

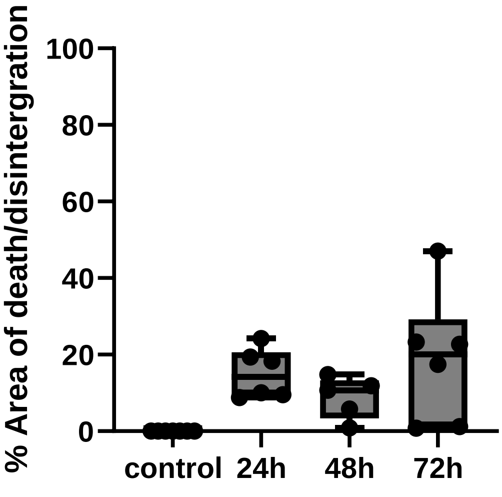

Supplement: Supplementary file 5 — Supplementary Information 5. [file 41598_2024_63170_MOESM5_ESM.pdf]
