# Supplementary material for: An optimised patient-derived explant platform for breast cancer reflects clinical responses to chemotherapy and antibody-directed therapy
Source: Sci Rep. 2024 Jun 4;14:12833. doi: 10.1038/s41598-024-63170-0 (PMC11150370; doi:10.1038/s41598-024-63170-0)
Supplement: Supplementary file 6 — Supplementary Information 6. [file 41598_2024_63170_MOESM6_ESM.pdf]

An optimised patient-derived explant platform for breast cancer reflects clinical responses to chemotherapy and antibody-directed therapy

Constantinos Demetriou[1], Naila Abid[1], Michael Butterworth[1], Larissa Lezina[1], Pavandeep Sandhu[1], Lynne Howells[1], Ian R Powley[1], James Howard Pringle[1], Zahirah Sidat[2], Omar Qassid[1,3], Dave Purnell[3], Monika Kaushik[4], Kaitlin Duckworth[4], Helen Hartshorn[4], Anne Thomas[1], Jacqui A Shaw[1], Marion MacFarlane[5,6]*, Catrin Pritchard[1]*, Gareth J Miles[1]*

Additional File 6

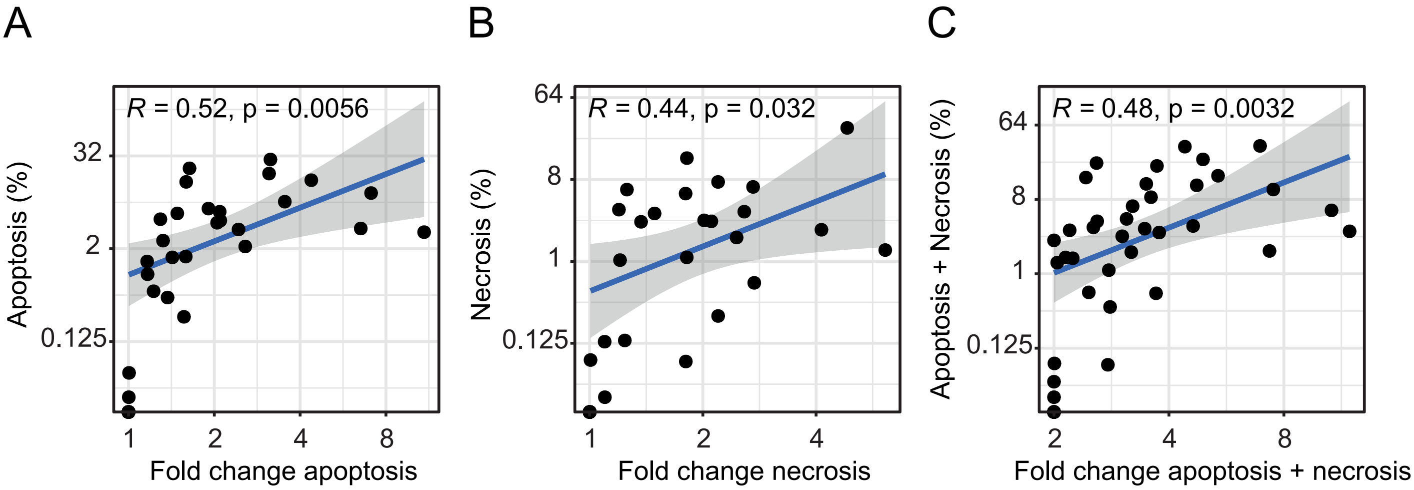

Additional File 6. Fold change apoptosis and necrosis relative to control versus percent apoptosis and necrosis relative to control in response to FET

A, correlation between tumour apoptosis in FET treated BC-PDEs after subtracting control apoptosis (%) and fold change apoptosis over control. **B,** correlation between area necrosis in FET treated samples after subtracting control necrosis and fold change necrosis over control. **C,** correlation between combined tumour apoptosis and necrosis in FET treated samples after subracting coltrol values and fold change tumour apoptosis and necrosis over control. A single dot represents the median value of respective values for al lPDEs derived from a give patient sample. Correlation was performed using the Pearson's rank test, where $p < 0.05$ is considered significant.
