# Supplementary material for: An optimised patient-derived explant platform for breast cancer reflects clinical responses to chemotherapy and antibody-directed therapy
Source: Sci Rep. 2024 Jun 4;14:12833. doi: 10.1038/s41598-024-63170-0 (PMC11150370; doi:10.1038/s41598-024-63170-0)

An optimised patient-derived explant platform for breast cancer reflects clinical responses to chemotherapy and antibody-directed therapy

Constantinos Demetriou[1], Naila Abid[1], Michael Butterworth[1], Larissa Lezina[1], Pavandeep Sandhu[1], Lynne Howells[1], Ian R Powley[1], James Howard Pringle[1], Zahirah Sidat[2], Omar Qassid[1,3], Dave Purnell[3], Monika Kaushik[4], Kaitlin Duckworth[4], Helen Hartshorn[4], Anne Thomas[1], Jacqui A Shaw[1], Marion MacFarlane[5,6]*, Catrin Pritchard[1]*, Gareth J Miles[1]*

Additional File 7. BC-PDE cell death responses to FET in the Stromal areas

A, FET-induced stromal apoptosis expressed as fold change over control across 46 BC-PDE samples. **B**, Correlation between FET-induced stromal apoptosis and FET-induced tumour apoptosis. **C**, Correlation between FET-induced stromal apoptosis and FET-induced combined tumour apoptosis and necrosis. A single dot represents the median values for all PDEs derived rom a given patient, and correlations are Pearson's rank test where $p < 0.05$ is considered significant.

Additional File 7

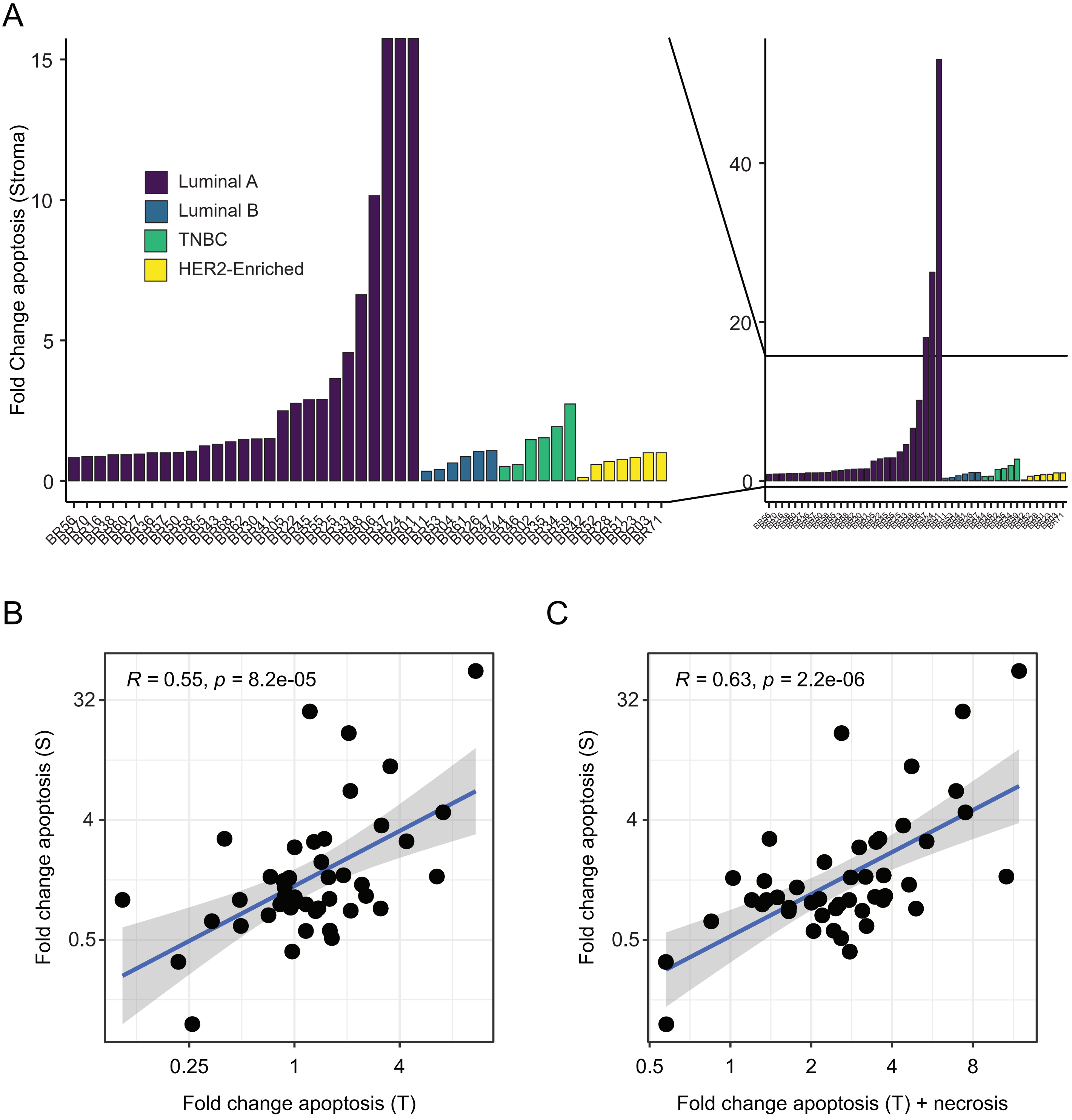

Supplement: Supplementary file 7 — Supplementary Information 7. [file 41598_2024_63170_MOESM7_ESM.pdf]
