# Supplementary material for: An optimised patient-derived explant platform for breast cancer reflects clinical responses to chemotherapy and antibody-directed therapy
Source: Sci Rep. 2024 Jun 4;14:12833. doi: 10.1038/s41598-024-63170-0 (PMC11150370; doi:10.1038/s41598-024-63170-0)

An optimised patient-derived explant platform for breast cancer reflects clinical responses to chemotherapy and antibody-directed therapy

Constantinos Demetriou[1], Naila Abid[1], Michael Butterworth[1], Larissa Lezina[1], Pavandeep Sandhu[1], Lynne Howells[1], Ian R Powley[1], James Howard Pringle[1], Zahirah Sidat[2], Omar Qassid[1,3], Dave Purnell[3], Monika Kaushik[4], Kaitlin Duckworth[4], Helen Hartshorn[4], Anne Thomas[1], Jacqui A Shaw[1], Marion MacFarlane[5,6]*, Catrin Pritchard[1]*, Gareth J Miles[1]*

Additional File 8: Ki67 and Geminin changes in BC-PDEs in response to FET

A, whole slide Ki67, cytokeratin and Geminin mIF-stained sections of BC-PDEs derived from 37 patients were digitised using the Vectra Polaris. Captured images were used to generate pseu-do-DAB images images of cytokeratin, Ki67 and Geminin using PhenoChart. Scale bar represents 20 µm. Images were then analysed in InForm to identify Geminin+ve and Ki67+ve cells. **B,** Correla-tion between the number of Ki67 positive cells and Geminin positive cells in BC-PDEs. Each dot represents a single explant, derived from either control or FET-treated samples. 560 BC-PDEs were used for this analysis **C,** Correlation between fold change Ki67 and fold change geminin with respect to control, in FET treated BC-PDEs, each dot represents the median values for all PDEs derived from a given patient. Correlations are Pearson's rank test where $p < 0.05$ is considered significant.

Additional File 8

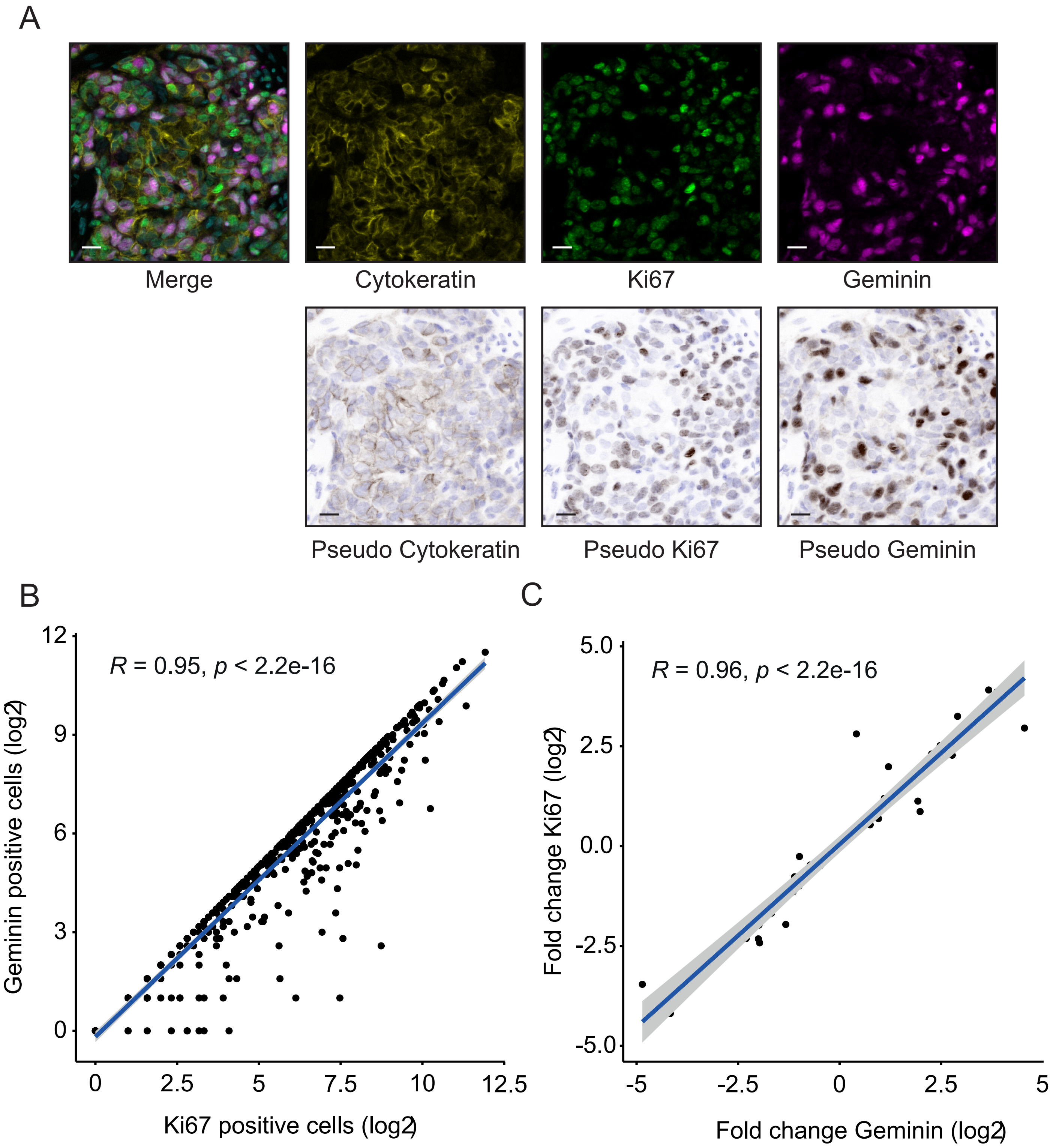

Supplement: Supplementary file 8 — Supplementary Information 8. [file 41598_2024_63170_MOESM8_ESM.pdf]
