# Supplementary material for: An optimised patient-derived explant platform for breast cancer reflects clinical responses to chemotherapy and antibody-directed therapy
Source: Sci Rep. 2024 Jun 4;14:12833. doi: 10.1038/s41598-024-63170-0 (PMC11150370; doi:10.1038/s41598-024-63170-0)

An optimised patient-derived explant platform for breast cancer reflects clinical responses to chemotherapy and antibody-directed therapy

Constantinos Demetriou[1], Naila Abid[1], Michael Butterworth[1], Larissa Lezina[1], Pavandeep Sandhu[1], Lynne Howells[1], Ian R Powley[1], James Howard Pringle[1], Zahirah Sidat[2], Omar Qassid[1,3], Dave Purnell[3], Monika Kaushik[4], Kaitlin Duckworth[4], Helen Hartshorn[4], Anne Thomas[1], Jacqui A Shaw[1], Marion MacFarlane[5,6]*, Catrin Pritchard[1]*, Gareth J Miles[1]*

Additional File 9: BC-PDE responses to FET + Trastzumab

40 BC-PDEs were treated with FET + Trastuzumab or FET treatment alone. tumour apoptosis and combined tumour apoptosis ane necrosis values in FET onlye treated BC-PDEs were subtracted from patient matched FET+Trastu-zumab treated BC-PDEs- to evaluate the additive effect of Trastuzumab on tumour cell killing above FET only treatment.

A, Percent change in tumour apoptosis of FET+ Trastuzumab treated BC-PDEs subtracting FET-induced tumour apoptosis related to HER2 status

B, Percent changin in combined tumour apoptosis and necrosis of FET + Trastuzumab treated BC-PDEs subtracting FET-induced tumour apoptosis and necrosis related to HER2 status.

Additional File 9

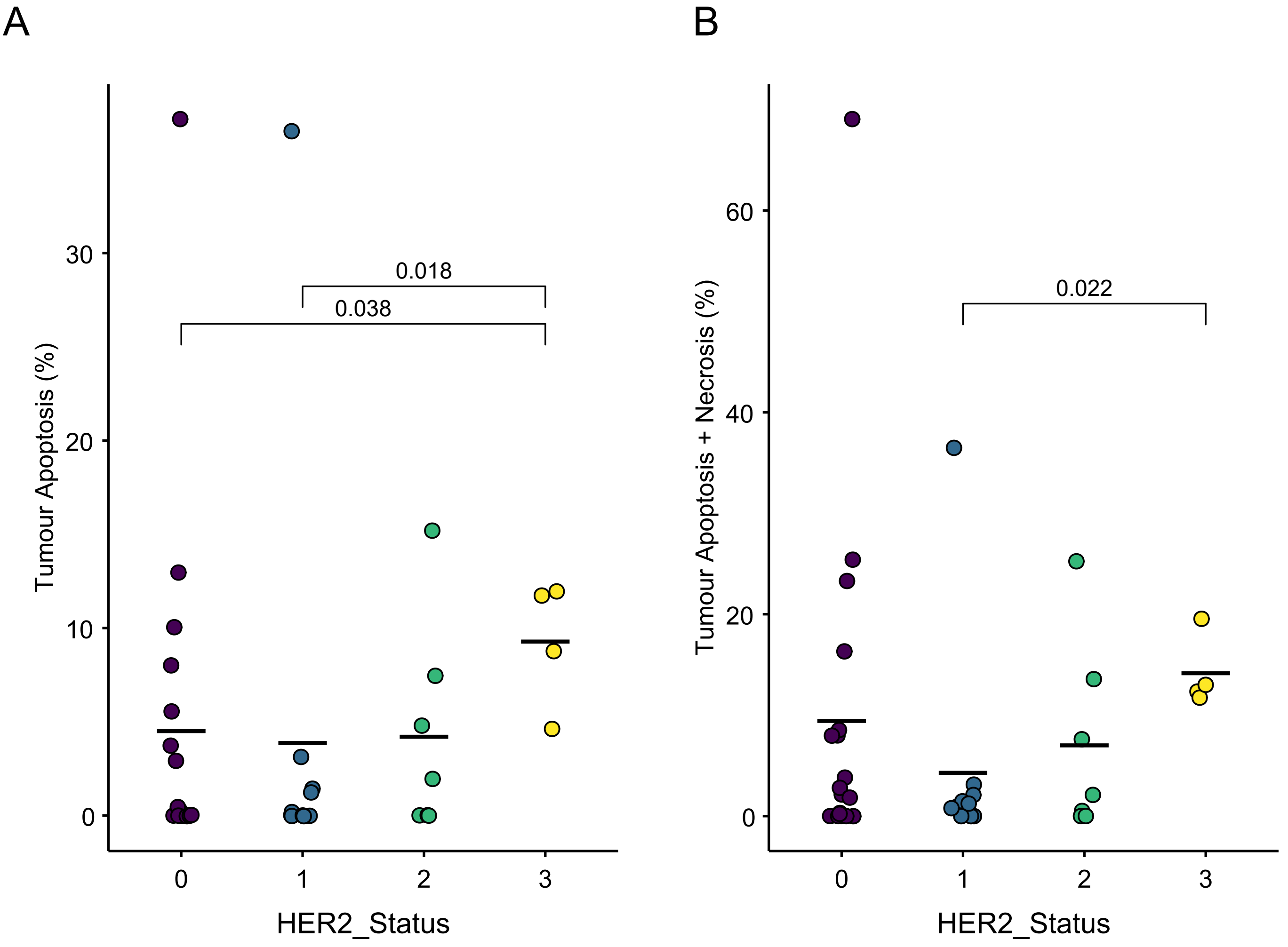

Supplement: Supplementary file 9 — Supplementary Information 9. [file 41598_2024_63170_MOESM9_ESM.pdf]
